# The Chemistry of the Defensive Secretions of Three Species of Millipedes in the Genus *Brachycybe*

**DOI:** 10.1007/s10886-024-01518-6

**Published:** 2024-06-10

**Authors:** Paige Banks, Emma M. Funkhouser, Angie M. Macias, Brian Lovett, Shelby Meador, Arden Hatch, H. Martin Garraffo, Kaitie C. Cartwright, Matt T. Kasson, Paul E. Marek, Tappey H. Jones, Emily Mevers

**Affiliations:** 1 Department of Chemistry, Virginia Tech, Blacksburg, VA 24061 USA; 2 Department of Chemistry, Virginia Military Institute, Lexington, VA 24450 USA; 3 Division of Plant and Soil Sciences, West Virginia University, Morgantown, WV 26506 USA; 4 Emerging Pests and Pathogens Research Unit, USDA ARS, Ithaca, NY 14853 USA; 5 National Institute of Diabetes and Digestive and Kidney Diseases, National Institutes of Health, Bethesda, MD 20892 USA; 6 National Institute of Standards and Technology, Gaithersburg, MD 20899 USA; 7 Department of Entomology, Virginia Tech, Blacksburg, VA 24061 USA

**Keywords:** Millipedes, Defensive secretions, Alkaloids, Structure elucidation

## Abstract

Millipedes have long been known to produce a diverse array of chemical defense agents that deter predation. These compounds, or their precursors, are stored in high concentration within glands (ozadenes) and are released upon disturbance. The subterclass Colobognatha contains four orders of millipedes, all of which are known to produce terpenoid alkaloids—spare the Siphonophorida that produce terpenes. Although these compounds represent some of the most structurally-intriguing millipede-derived natural products, they are the least studied class of millipede defensive secretions. Here, we describe the chemistry of millipede defensive secretions from three species of *Brachycybe*: *Brachycybe producta*, *Brachycybe petasata*, and *Brachycybe rosea*. Chemical investigations using mass spectrometry-based metabolomics, chemical synthesis, and 2D NMR led to the identification of five alkaloids, three of which are new to the literature. All identified compounds are monoterpene alkaloids with the new compounds representing indolizidine (i.e. hydrogosodesmine) and quinolizidine alkaloids (i.e. homogosodesmine and homo-hydrogosodesmine). The chemical diversity of these compounds tracks the known species phylogeny of this genus, rather than the geographical proximity of the species. The indolizidines and quinolizidines are produced by non-sympatric sister species, *B. producta* and *B. petasata*, while deoxybuzonamine is produced by another set of non-sympatric sister species, *B. rosea* and *Brachycybe lecontii*. The fidelity between the chemical diversity and phylogeny strongly suggests that millipedes generate these complex defensive agents *de novo* and begins to provide insights into the evolution of their biochemical pathways.

**Supplementary Information:**

The online version contains supplementary material available at 10.1007/s10886-024-01518-6.

## Introduction

Millipedes (class Diplopoda) produce a myriad of defensive chemicals, including hydrogen cyanide, oxidized aromatics (e.g., benzoquinones), and alkaloids (e.g., quinazolinone and terpene alkaloids) (Shear 2015). These compounds are stored in high concentrations in ozadenes (or rapidly generated from biological inert precursors) and the millipedes release the defensive agents when disturbed (Eisner et al. 1978; Carrel 1984). Some other millipedes use chemical secretions for defense against parasites and microbes, protection during the process of molting, and crypsis and background matching (Youngsteadt 2008; Shear 2008; Marek et al. 2012; Ilić et al. 2019; Shear and Marek 2022). 385-million-year-old Devonian fossil millipedes show the first evidence of chemical defenses on land from the presence of ozopores (openings of the ozadenes) that line the length of the fossilized body (Wilson 2006). Plausible origins of chemical defenses in millipedes are even older and their exclusive presence in the subclass Chilognatha indicates a Silurian origin 426 million years ago (Rodriguez et al. 2018). The evolutionary development of millipede defense glands appears similar to other arthropods in which cuticular invaginations became lined with glands to produce chemicals. Over evolutionary time, glands diversified into three or four types: (1) bilateral single-chambered glands of Juliformia and Nematophora, (2) median Y-shaped glands of Glomerida, and (3) bilateral bipartite glands of Polydesmida (Minelli 2013; Shear 2015). The defense glands of *Brachycybe lecontii* were described as long slender tubes, indicative of a fourth gland morphotype (Wood 1864; Eisner et al. 1978). However, later authors showed that the gland architecture of *B. lecontii* is tear-drop-shaped and more voluminous than previously described and consists of a single chamber (akin to a type 1 glands), containing the defense secretions, connected to a duct leading from the ozadene to the ozopore (Wong et al. 2020a). Bipartite and Y-shaped glands are ostensibly derived from single-chambered glands, but gland morphotype is known from a limited set of taxa, and a fully resolved phylogeny of millipedes with representatives of each order is not yet available as a context to address questions about gland morphological evolution.

**Fig. 1:**
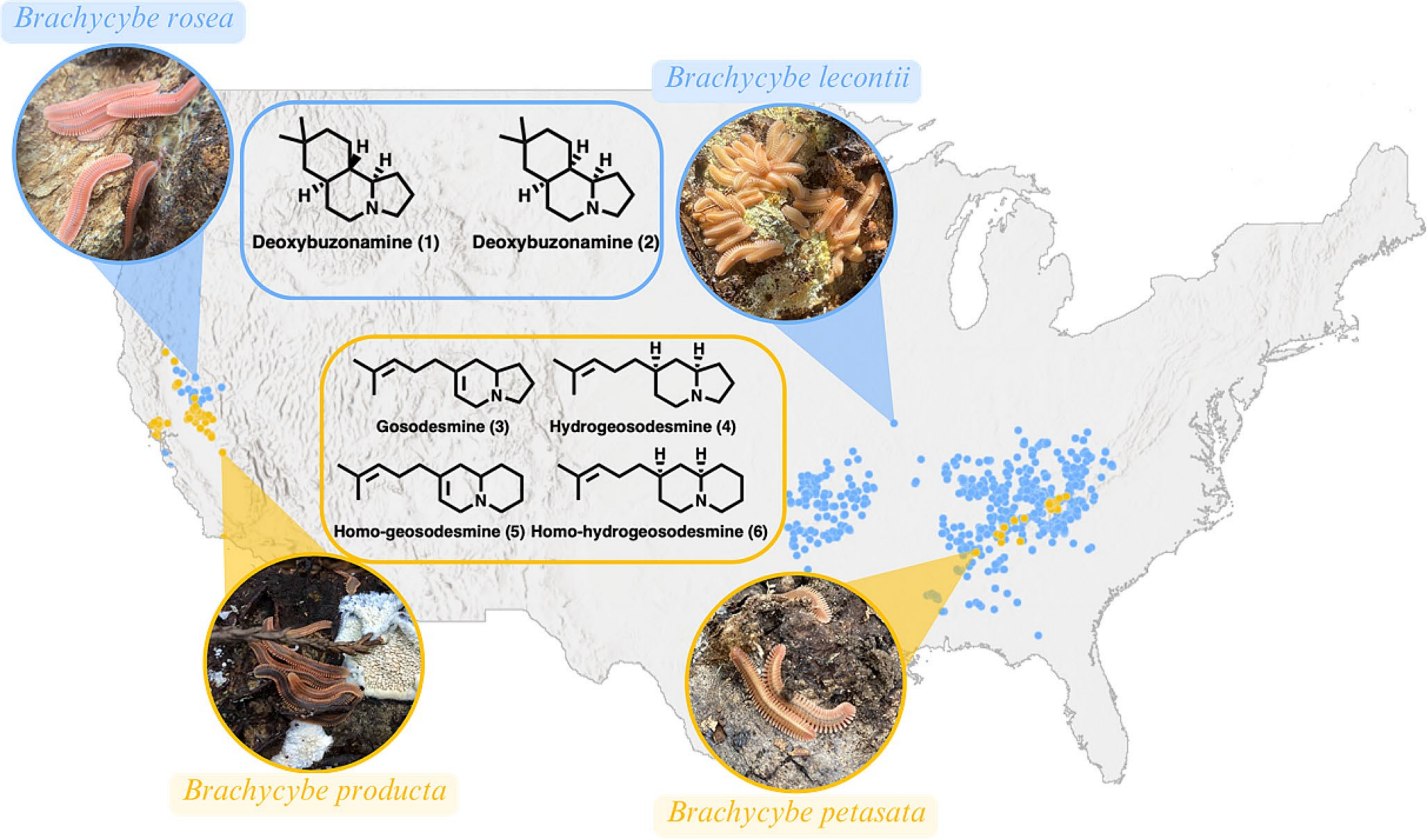
(**A**) Geographic distribution of *Brachycybe producta, Brachycybe petasata, Brachycybe lecontii* and *Brachycybe rosea* with their associated defensive agents

Of the known defensive chemicals, the alkaloids are the most structurally intriguing and least studied. Structurally, these compounds can be classified into two groups: the quinazolinone alkaloids (e.g., glomerin and homoglomerin) and terpenoid alkaloids (e.g., polyzonimine and buzonamine) (Smolanoff et al. 1975; Wood et al. 2000; Shear et al. 2011). The quinazolinone alkaloids are produced by pill millipedes from the order Glomerida and appear to be derived from benzoic acid (shikimate pathway) and hydrogen cyanide (Dewick 2009). Therefore, these structures represent a hybrid between the oxidized aromatic and hydrogen cyanide products, making them pseudoalkaloids. Conversely, the terpenoid alkaloids are produced by a single subterclass (Table 1), the Colobognatha (fungus-feeding millipedes). This subterclass is composed of four orders (Platydesmida, Polyzoniida, Siphonocryptida and Siphonophorida) and all are known to produce simple monoterpenes (pinenes and limonene), along with various heterocyclic terpenoid alkaloids [Fig. 1; e.g. deoxybuzonamines (**1** and **2**), and gosodesmine (**3**)] (Shear 2015; Hassler et al. 2020; Jones et al. 2022). However, the terpenoid alkaloids can be further separated into two structural classes, the spirocyclic (e.g., polyzonimine and nitropolyzonamine) and heterocyclic alkaloids (e.g., buzonamine and gosodesmine) (Meinwald et al. 1975; Smolanoff et al. 1975; Wood et al. 2000; Hassler et al. 2020). Both classes incorporate a monoterpene, as evident by the *gem*-dimethyl moiety. For the heterocyclic alkaloids, the nitrogen is likely derived from an amino acid, either proline or ornithine, making these metabolites true alkaloids (Dewick 2009). Although the alkaloids are less studied than the other classes of defensive secretions, there are recent reports describing new compounds representing both the heterocyclic and spirocyclic families of alkaloids. This includes the discovery of deoxybuzonamine isomers and acetylated 3-hydroxynitropolyzonamine (Jones et al. 2022; Kunert et al. 2023). Interestingly, the spirocyclic alkaloids are known to be sequestered by neotropical frogs for defensive purposes (Kuwahara et al. 2007).

Members of the millipede order Platydesmida have intriguing qualities, such as egg brooding, and other quasisocial traits, and are almost exclusively associated with various fungal taxa, which some consume in unusual circular social aggregations called pinwheels (Macias et al. 2019; Wong et al. 2020b). All of the species of the Platydesmida with characterized chemical secretions, and its evolutionary sister Polyzoniida (Rodriguez et al. 2018), have disparate heterocyclic terpenoid alkaloids, including *Brachycybe lecontii* from the eastern U.S. that produces compounds **1** and **2**, and *Gosodesmus claremontus* from California and Oregon that produces **3** (Hassler et al. 2020; Jones et al. 2022) (Fig. 1). Interestingly, co-occurrence of species from the same genera does not correlate with phylogenetic relationship. For example, *B. lecontii* is sympatric with *Brachycybe petasata*, but *B. lecontii’s* closest evolutionary sister is *Brachycybe nodulosa*, endemic to Japan (Brewer et al. 2012). To date, it is unknown how the chemical diversity of the defensive secretions relates to the reported phylogenetic relationship. Herein, we investigated the composition of the defensive secretions of three unstudied species of *Brachycybe* – *B. petasata*, *B. producta*, and *B. rosea* – to gain a better understanding of the relationship between phylogeny and defensive secretions.

**Table 1:** Millipede subterclass Colobognatha chemistry (excluding the order Siphonophoride)

Millipede	Compound	Source
*Rhinotus purpureus*	O-methyloxime 236	Saporito et al. 2003
*Buzonium crassipes*	Buzonamine, β-pinene, limonene	Wood et al. 2000
*Polyzonium rosalbum*	Polyzonimine, nitropolyzonamine	Meinwald et al. 1975; Smolanoff et al. 1975
*Petaserpes cryptocephalus*	Polyzonimine, nitro-polyzonamine	Meinwald et al. 1975; Shear 2015
*Polyzonium germanicum*	Polyzonimine, nitro-polyzonamine, acetylated 3-hydroxy-nitro-polyzonamine and three isomers	Kunert et al. 2023
*Gosodesmus claremontus*	Buzonamine	Hassler et al. 2020
*Brachycybe lecontii*	Deoxybuzonamine isomers	Jones et al. 2022
*Kiusiozonium okai*	Polyzonimine, nitropolyzonamine, O- methyloxime 236	Kuwahara et al. 2007
*Hirudicryptus canariensis*	O-methyloxime 236	Shear 2015
*Brachycybe rosea*	Deoxybuzonamine	This study
*Brachycybe petasata*	Gosodesmine, homogosodesmine, hydrogosodesmine, homohydrogosodesmine	This study
*Brachycybe producta*	Gosodesmine, homogosodesmine, hydrogosodesmine, homo-hydrogosodesmine	This study

## Methods and Materials

### General Experimental Procedures

NMR spectra were recorded with deuterated DMSO with the residual solvent peak as an internal standard (δ_C_ 39.5, δ_H_ 2.50) on Bruker Avance III 600 MHz instrument equipped with a triple resonance inverse (CP-TCI) Prodigy N2 cooled CryoProbe (600 and 150 MHz for ^1^H and ^13^C NMR, respectively) and a JEOL 400 Mhz NMR spectrometer. GCMS was carried out in the electron impact (EI) mode using a Shimadzu QP-2020. LR-LCMS data was obtained using an Agilent 1200 series HPLC system equipped with a photo-diode array detector and a Thermo LTQ mass spectrometer. HRESIMS was carried out using a Shimadzu LC-q-TOF Mass Spectrometer equipped with a HPLC system. HPLC purifications were carried out using Agilent 1200 series or 1260 Infinity II HPLC systems (Agilent Technologies) equipped with a photodiode array detector. All solvents were of HPLC quality. Optical rotation was measured using a JASCO P-2000 polarimeter.

### Millipede Collections

The millipedes were collected at the following locations. (1) *B. petasata*, Haywood Co., North Carolina, Balsam Mountain Campground, nature trail, 35.56764°N, -83.17651°W, Elev. 1622 m, 27 September 2021 (PEM-2021-007, habitat of hemlock, maple, birch/beech); (2) *B. petasata*, Sevier Co., Tennessee, Great Smoky Mountains, Mt. LeConte, Alum Cave Trail, 35.63998°N, -83.44023°W, Elev. 1409 m, 28 September 2021, (PEM-2021-008, habitat *Rhododendron* cove); (3) *B. petasata*, Dade Co., Georgia, Cloudland Canyon State Park, forested area along roadside across from the visitor center, 34.817213°N, -85.487654°W, Elev. 563 m, 15 March, 2021 (MTK-GA-21-BPE13); (4) *B. producta*, Marin Co., California, Lake Lagunitas, Marin Municipal Water District, adjacent to Lake Lagunitas Loop Trail 37.946577°N, -122.597678°W, Elev. 247 m, 5 December 2021 (MTK-CA-21-5); (5) *B. producta*, Marin Co., California, Lake Lagunitas, Marin Municipal Water District, adjacent to Indian Fire Road, Eldridge Grade on Mount Tamalpais 37.934565°N, -122.572577°W, Elev. 418 m, 5 December 2021 (MTK-CA-21-6); (6) *B. rosea*, El Dorado Co., California, El Dorado Irrigation District, Sly Park Recreation Area, forested area adjacent to Miwok trail 38.732112°N, -120.559085°W, Elev. 1083 m, 11 December 2021 (MTK-CA-21-12); (7) *B. rosea*, San Mateo Co., California, Edgewood Park and Nature Preserve, edge of ravine next to restrooms, 37.472201°N, -122.278708°W, Elev. 83 m, 4 December 2021 (MTK-CA-21-3); and (8) *B. rosea*, Tahoe National Forest, Upper Carlton Campground, 39.520737°N, -120.996210°W, Elev. 712 m, 10 December 2021 (MTK-CA-21-13). All collections were placed in small vials with a few mL of methanol for preservation and immediate extraction of the defensive secretions. Voucher specimens have been deposited as natural history specimens in the Virginia Tech Insect Collection (https://collection.ento.vt.edu/).

### General GCMS Method Used for all Analyses

GCMS analyses were carried out in the electron impact (EI) mode using a Shimadzu QP-2020 equipped with an RTX-5 column (30 m x 0.25 mm i.d. column) programmed from 60 °C to 250 °C changing at a rate of 10 °C/min and holding at 250 °C for 12 min.

### GCMS Analysis of Collected Millipedes Crude Extracts

An aliquot of each crude extract from distinct millipede collections were analyzed using the general GCMS method (described above). All *B. rosea* collections had a prominent peak matching the retention time (17.7 min) and fragmentation as an authentic sample of deoxybuzonamine (**1**). EIMS *m/z* (rel %) 207 (45, M^+^), 206 (100), 192 (16), 178 (20), 136 (18), 97 (25), 96 (36), 84 (69), 83 (45). *B. petasata* and *B. producta* collections had variable prominent peaks at 17.6 (**4**), 18.0 (**3**), 19.0 (**6**), and 19.4 (**5**) min, which are summarized in Table 2. The peak at 18.001 matched the retention time and fragmentation as an authentic sample of gosodesmine (**3**). EIMS *m/z* (rel %) 205 (17, M^+^), 204 (35), 136 (50), 122 (22), 93 (82), 70 (100), 53 (10), 41 (62).

### Chemical Derivatization of *B. petasata* and *B. producta* Crude Extracts

An aliquot of a representative crude extract from both *B. petasata* and *B. producta* millipedes was derivatized using microhydrogenation. A slow stream of hydrogen was bubbled through the original extract, ca. 50 µL, containing a few milligrams of PtO_2_ until the catalyst turned black. The supernatant was immediately subjected to GCMS analysis using the general method described above. Hydrogenation of extracts of both species gave two isomers of both 7-(4-methylpentyl)indolizidine (**7**) and 2-(4-methylpentylquinolizidine (**8**) in a 1:2 ratio (synthesis described below) (Hassler et al. 2020). In addition, the alkaloids in one collection of *B. producta* were hydrated by treating a small portion of the extract with 5% HCl in MeOH for 1 h at rt, at which point the mixture was carefully made basic with solid K_2_CO_3_ and extracted with 200 µL of diethyl ether. Analysis by GCMS showed the original **3** and **4** along with two new peaks with M^+^ water adducts of **5** and **6**.

### Synthesis of 2-(4-Methylpentyl)-quinolizidine (8)

A 1.6 mol/L solution of *n*-butyllithium in hexane (1.0 mL) was added dropwise to a slurry containing 0.55 g (1.3 mmol) of 4-methylpentyltriphenylphosphonium bromide in 10 mL of THF under argon at 0 ^o^C (Dickschat et al. 2005). The mixture was stirred for 0.5 h followed by the dropwise addition of 0.17 g (1.1 mmol) of 2-quinolizidone in 1 mL of THF and allowed to come to rt overnight (Hermet et al. 2003). The mixture was filtered through celite and the solvent removed under pressure. The residue was triturated with 15 mL of anhydrous ether and filtered through a silica gel plug to provide 0.15 g of a 1:1 mixture of the starting 2-quinolizidone and a 1:1 mixture of (*E* and *Z*) isomers of 2-(4-methylpentylidine)octahydroquinolizidine with identical mass spectra: MS *m/z* (rel %) 221 (40, M^+^), 220 (43), 206 (13), 178 (20), 165 (12), 164 (75), 151 (20), 150 (100), 136 (48), 122 (10), 97 (53), 96 (35), 84 (35). This material was taken up in MeOH and treated with ca. 10 mg of PtO_2_ and a slow stream of hydrogen was bubbled through it until the catalyst turned black. After 20 min, the sample was filtered and the mixture analyzed on GCMS, which showed the presence of two isomers of **8** in a 12:1 ratio having identical mass spectra. Compound **8** was an amorphous colorless solid; EIMS *m/z* (rel %) 223 (43, M^+^), 222 (82), 208 (25), 194 (7), 180 (57), 166 (11), 152 (14), 139 (10), 138 (100), 110 (46), 97 (57), 96 (14), 84 (16), 83 (16), 82 (18).

### Synthesis of Homogosodesmine (5)

A solution containing 0.23 g (1.08 mmol) of piperidine ketone (**9**), 0.21 g of diethyl phosphonoacetic acid, and 0.21 g of EDCI in 5 mL of DMF was stirred overnight at rt under an argon atmosphere. The mixture was diluted with 25 mL of water and extracted 3 × 50 mL of diethyl ether. The combined ether extracts were dried over anhydrous MgSO_4_, filtered and concentrated to provide a crude mixture containing a 1:4 mixture of the diethyl phosphonoacetamide (**10**) (M = 387) and lactam **11** (M = 233). EIMS *m/z* (rel %) 387 (13, M^+^), 262 (51), 208 (35), 140 (20), 123 (10), 98 (52), 84 (100), 69 (7). The residue was taken up in 5 mL of anhydrous acetonitrile (ACN) and treated with 0.15 g of DBU (0.973 mmol) and 50 mg of LiCl (1.18 mmol) and the solution was stirred overnight at rt. The solvent was removed under reduced pressure, and the residue was taken up in 20 mL of diethyl ether and extracted with 5 mL of water. The ether solution was passed through a short plug of silica gel and the solvent was removed to provide 0.25 g of nearly pure **11**, which was used as is in the next reaction. EIMS *m/z* 233 (19, M^+^), 218 (5), 205 (9), 190 (7), 176 (3), 166(12), 165(100), 164 (44), 152 (12), 84 (30), 69 (16), 41, (14). A solution containing 0.2 g of lactam **11** in 2 mL of diethyl ether was added to a mixture containing 0.12 g of LiAlH_4_ in 40 mL of diethyl ether and stirred for 3 h at rt. The reaction was quenched by sequential addition of 5–6 drops of water, 5–6 drops of 10% NaOH, and 10 drops of water, filtered, and solvent removed by reduced pressure. This yielded 0.18 g of **5** whose mass spectrum and retention times matched those of the quinolizidine natural product from *B. producta.* Compound **5** was an amorphous colorless solid, ^1^H and ^13^C NMR: See Table S1; EIMS *m/z* (rel %) 219 (48, M^+^), 218 (70), 204 (11), 191 (13), 176 (24), 162 (20), 151 (24), 150 (100), 149 (33), 136 (50), 134 (15), 93 (21), 84 (78), 69 (22), 55 (13), 41 (28); HRMS (ESI) calc for C_15_H_26_N^+^ 220.2060, found [M + H]^+^ 220.2058, Δ 0.75 ppm.

### Synthesis of 7-(4-Hydroxy-4-methylpentyl)-indolizidine (18)

A 1 mol/L solution of LiHMDS (2.2 mL) in hexanes was added slowly to a solution containing 0.5 g (2 mmol) of triethyl-4-phosphonocrotonate in 10 mL of THF at 0 ^o^C under an argon atmosphere. After 0.5 h a solution containing 0.28 g (2.01 mmol) of 7-indolizidone (**12**) (Hermet et al. 2003) in 2 mL of THF was added and the mixture was stirred overnight. The mixture was diluted with 70 mL of diethyl ether and stirred with 5 mL of saturated NH_4_Cl (aq). The layers were separated, and the organic layer was dried over anhydrous K_2_CO_3_ and the solvent was removed under reduced pressure to provide 0.22 g of a mixture of geometric isomers of **14**. EIMS *m/z* 235 (62, M^+^), 206 (22), 190 (18), 162 (29), 176 (28), 162 (34), 160 (11), 83 (100), 81 (53), 70 (24). The crude mixture was taken up in 20 mL of EtOH and treated with 1 mL of 10% HCl, and hydrogenated over 40 mg of 10% Pd/C at 3 atm pressure for 45 min. The mixture was filtered through celite and after removal of the solvent partitioned between diethyl ether and 10% NaOH (aq). The organic layer was dried over anhydrous K_2_CO_3_, the solvent was removed from the ether solution to provide 0.19 g of the saturated amine ester (**16**) as a 2:1 mixture of isomers. EIMS *m/z* 239 (40, M^+^), 238 (100), 210 (27), 194 (36), 152 (31), 138 (24), 124 (91), 97 (31), 96 (80), 83 (33). This product was taken up in 10 mL of THF and treated with 2 mL of 3 mol/L MeMgCl in THF and allowed to stir overnight. The mixture was diluted with diethyl ether and treated with 10 mL of saturated NH_4_Cl (aq). The organic layer was separated, dried over anhydrous K_2_CO_3_ and the solvent was removed to provide 0.11 g of **16** as a 2:1 mixture of isomers with identical mass spectra. The GC retention time and the mass spectrum of the first eluting isomer matched that of the hydrated natural product (**18**). Compound **18** was an amorphous colorless solid, ^1^H and ^13^C NMR: See **Table S2** (major stereoisomer) and **S3** (minor stereoisomer); EIMS *m/z* 225 (44, M^+^), 224 (100), 210 (676), 166 (100), 138 (44), 124 (81), 97 (23), 96 (60), 83 (26), 59 (14), 55 (10). HRMS (ESI) calc for C_14_H_28_NO^+^ 226.2165, found [M + H]^+^ 226.2166, Δ 0.13 ppm.

### Synthesis of 2-(4-Hydroxy-4-methylpentyl)-quinolizidine (19)

This compound was prepared using the same method described for **18**, except it started with 2-quinolizidone (**13**). A 1 mol/L solution of LiHMDS (2.2 mL) in hexanes was added slowly to a solution containing 0.5 g (2 mmol) of triethyl-4-phosphonocrotonate in 10 mL of THF at 0 ^o^C under an argon atmosphere. After 0.5 h a solution containing 0.3 g (1.96 mmol) of 2-quinolizidone (Hermet et al. 2003) in 2 mL of THF was added and the mixture was stirred overnight. The mixture was diluted with 70 mL of diethyl ether and stirred with 5 mL of saturated NH_4_Cl (aq). The layers were separated, and the organic layer was dried over anhydrous K_2_CO_3_ and the solvent was removed under reduced pressure to provide a mixture of geometric isomers of **15**. EIMS *m/z* 249 (70, M^+^), 234 (36), 220 (20), 204 (20), 176 (28), 162 (34), 136 (22), 97 (100), 96 (99), 69 (22). The crude mixture was taken up in 20 mL of EtOH treated with 1 mL of 10% HCl, and hydrogenated over 40 mg of 10% Pd/C at 3 atm pressure for 45 min. The mixture was filtered through celite and after removal of the solvent partitioned between diethyl ether and 10% NaOH (aq). After drying over anhydrous K_2_CO_3_ the solvent was removed from the ether solution to provide the saturated amine ester **17**. EIMS *m/z* 253 (35, M^+^), 252 (67), 238 (11), 224 (23), 210 (11), 208 (48), 106 (47), 152 (30), 138 (100), 110 (67), 97 (77), 82 (41). This product was taken up in 10 mL of THF and treated with 2 mL of 3 mol/L MeMgCl in THF and allowed to stir overnight. The mixture was diluted with diethyl ether and treated with 10 mL of saturated NH_4_Cl (aq). The organic layer was separated, dried over anhydrous K_2_CO_3_ and the solvent was removed to provide 0.65 g of **19** as a 3:1 mixture of isomers with identical mass spectra. The GC retention time and the mass spectrum of the first eluting isomer matched that of the hydrated natural product (**19**). Compound **19** was an amorphous colorless solid; ^1^H and ^13^C NMR: See Table S4 (major stereoisomer) and S5 (minor stereoisomer); EIMS *m/z* 239 (20, M^+^), 238 (42), 224 (56), 180 (100), 152 (18), 138 (67), 110 (33), 97 (38), 83 (22), 59 (13), 55 (20); HRMS (ESI) calc for C_15_H_30_NO^+^ 240.2322, found [M + H]^+^ 240.2319, Δ 1.3 ppm.

### Extraction and Isolation of homogosodesmine (5) and homohydrogosodesmine (6) from *B. producta*

The millipedes were extracted using MeOH and dried down. The extract was then resuspended in 1:1 ACN/H_2_O and separated using reverse-phase high performance liquid chromatography (RP-HPLC). The mobile phase was a mixture of ACN and H_2_O with 0.1% formic acid, using a Phenomenex 4 μm Hydro semi-preparative column (250 × 10 mm). The flow was set to 3 mL/min with a 5 min isocratic hold at 20% ACN/H_2_O followed by a linear gradient to 47% ACN/H_2_O over 29 min to yield pure hydrogosodesmine (**4**) and homo-hydrogeosodesmine (**6**).

### Hydrogosodesmine (4)

Amorphous solid; [α]_D_ 3.75 (MeOH); ^1^H and ^13^C NMR: See Table S6; HRMS (ESI) calc for C_14_H_26_N^+^ 208.2060, found [M + H]^+^ 208.2061, Δ0.55 ppm.

### Hydro-homogosodesmine (6)

Amorphous solid; [α]_D_ 7.50 (MeOH); ^1^H and ^13^C NMR: See Table S7; HRMS (ESI) calc for C_15_H_28_N^+^ 222.2216, found [M + H]^+^ 222.2219, Δ1.3 ppm.

## Results

Initial examination of methanol extracts from the collections of millipedes revealed that *B. petasata* and *B. producta* produce four alkaloids, while *B. rosea* has only one alkaloid that is dissimilar from the other two millipedes (Table 2, **Fig. **S1- S3). There is a bit of variability in the abundance of some of the alkaloids, but this is likely resulting from the size of the collections. The sole alkaloid peak in the *B. rosea* extracts matched both the retention time (17.7 min) and fragmentation of one of the previously identified deoxybuzonamine isomers (**1**) originally isolated from *B. lecontii* (Fig. S4) (Jones et al. 2022). The major components in the *B. petasata* and *B. lecontii* extracts were separated by 14 Da [*B. petasata* - M^+^ 205 (**3**) and 219 (**5**); *B. producta* - M^+^ 207 (**4**) and 221 (**6**)] and the difference between the major components of the distinct millipede species was 2 Da. In *B. petasata* extracts, the first eluting alkaloid (M^+^ 205) had an identical mass spectrum and retention time with an authentic sample of gosodesmine (**3**) (Hassler et al. 2020). The other major alkaloids had no matches to known natural products; however, analysis of their fragmentation suggested they were structurally related to **3**.

**Table 2:** Percentage of alkaloid detected in different collections

Collection/ site	Species	M^+^ (compound)/Retention index				
		221 (1)/1419	205 (**3**)/1414	207 (**4**)/1446	219 (**5**)/1518	221 (**6**)/1550
MTK-GA-21-BPE13	*B. petasata*	n.d.	48	n.d.	49	trace
PEM-2021-007	*B. petasata*	n.d.	21	trace	19	59
PEM-2021-008	*B. petasata*	n.d.	7	37	49	20
MTK-CA-21-05	*B. producta*	n.d.	1.5	52	trace	48
MTK-CA-21-06	*B. producta*	n.d.	trace	43	trace	57
AH-CA-23-11	*B. producta*	n.d.	15	34	trace	51
MTK-CA-21-12	*B. rosea*	100	n.d.	n.d.	n.d.	n.d.
MTK-CA-21-13	*B. rosea*	100	n.d.	n.d.	n.d.	n.d.
MTK-CA-21-03	*B. rosea*	100	n.d.	n.d.	n.d.	n.d.
Dickson Co., TN^**1**^	*B. lecontii*	100	n.d.	n.d.	n.d.	n.d.

^1^Previously reported (Jones et al. 2022), trace = < 1% area

**Fig. 2:**
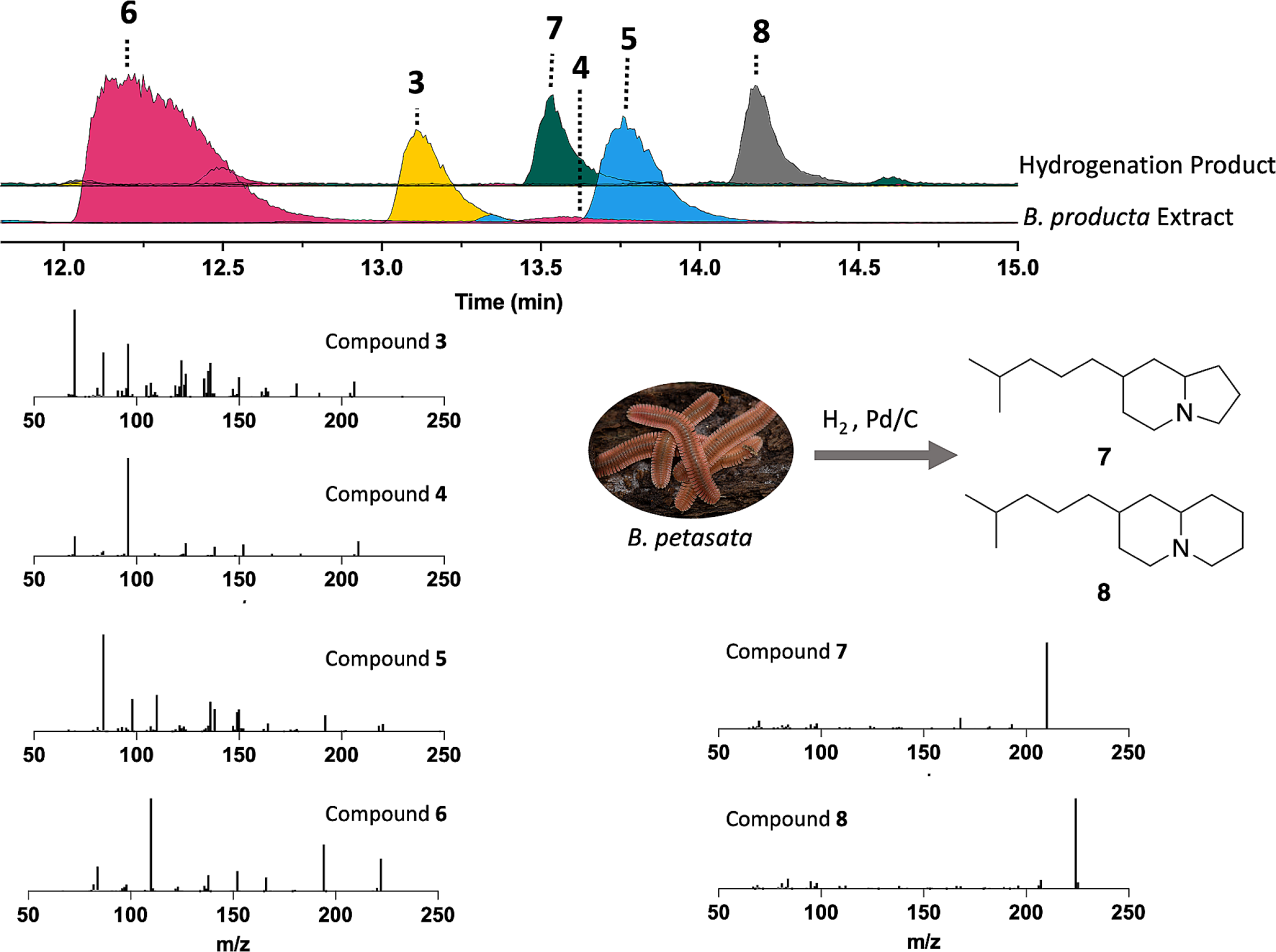
Comparison of extracted ion chromatograms (EICs) of the unreacted *B. producta* extract (PEM 2021-007) and hydrogenation product. Bottom chromatogram contain EICs of **3**–**6** (206, 208, 220, and 222 *m/z*), while the top chromatogram contains the same EICs plus **7** and **8** (210 and 224 *m/z*). The ion counts for the EICs associated with the crude extract were divided by an order of magnitude to scale to the hydrogenation reaction mixture. Spectra below chromatograms are LTQ tandem MS fragmentation patterns (35 eV) for compounds **3**–**8**

To understand the structural features of the new metabolites, the crude extracts were chemically derivatized and then analyzed by GCMS. First, it was hypothesized that the mass difference of 2 Da between the major compounds in *B. petasata* (**3** and **5**) and *B. producta* (**4** and **6**) is indicative of a loss of a degree of unsaturation—either the elimination of one of the olefins or a loss of one of the rings. The former is a typical post-modification event therefore this was believed to be more likely. So, a small aliquot of representative samples of each crude extract were treated with hydrogenation conditions to reduce all olefins. Subsequent GCMS analysis revealed the presence of the same two saturated amines in both derivatized crude extracts, including the known 7-(4-methylpentyl)-indolizidine (**7**) and a metabolite that was 14 Da larger (Fig. S5 and S6). We suspected the larger metabolite was likely 2-(4-methylpentyl)-quinolizidine (**8**). Synthesis of **8** was accomplished using a similar approach to the method previously reported for **7** but started from 2-quinolizidone. The synthetic material possessed the same GC fragmentation and retention time, thus confirming the carbon skeleton of the three new analogs, **4**–**6** (Fig. S7 and S8) (Hermet et al. 2003; Hassler et al. 2020). Compounds **3** and **4** incorporate indolizidine rings, and **5** and **6** incorporate quinolizidine rings (Fig. 2).

**Scheme 1:**
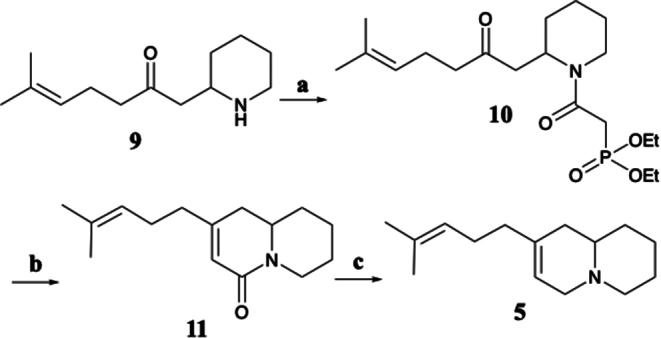
Synthesis of homogosodesmine (**5**). (**a**) EDCI, diethylphosphono acetic acid, DMF, 16 h, rt; (**b**) DBU, LiCl, ACN, 16 h, rt; (**c**) LiAlH_4_, diethyl ether, 3 h, rt

Compound **5** was hypothesized to be structurally similar to **3** but contain a quinolizidine rather than indolizidine ring. To confirm this, we attempted a total synthesis (Scheme 1), which began by carbodiimide coupling of 6-methyl-1-(piperidin-2-yl)hept-5-en-2-one (**9**) with diethylphosphonoacetic acid to yield **10** (Monaco et al. 2011). This was followed by an intramolecular Horner-Wadsworth-Emmons cyclization that cleanly provided 2-(4-methylpent-3-en-1-yl)-7,8,9,9a-tetrahydro-1*H*-quinolizin-4(6*H*)-one (**11**) (Nangia and Prasuna 1996). Subsequent treatment with lithium aluminum hydride to reduce the lactam gave what was predicted to be **5**, which we have named homogosodesmine. GCMS comparison of the synthetically derived material with the crude extract confirmed the structure of **5** as both the mass spectrum and GC retention time matched (Fig. S9 – S16; Table **S1**). Additionally, catalytic hydrogenation of a sample of synthetic **5** provided a mixture of isomers of **8** whose mass spectra and GC retention times were identical to those obtained from the hydrogenation of the millipede extracts.

Compounds **4** and **6** are close structural analogs of **3** and **5**, respectively, but based on the hydrogenation experiment they have only one olefin. Both alkaloids showed a loss of 85 Da in their mass spectra, which is typical of a loss of C_6_H_13_. This loss was observed in the mass spectra of **7** and **8** and represented the loss of the saturated side chain from the heterocyclic core, so initially we hypothesized that the olefin was within the ring systems. Fortuitously, treatment of a portion of the crude extract with dilute HCl (aq) and subsequent neutralization resulted in the addition of water (Fig. S17). The mass spectra of the hydrate adduct showed a loss of methyl (15 Da), intense ions for the loss of C_3_H_7_O fragment (59 Da) as well as the corresponding fragment at 59 *m/z* that would be expected from a dimethyl tertiary alcohol (Fig. S18). Thus, indicating that the olefin in **4** and **6** is on the sidechain opposed to within the bicyclic system. Since *B. petasata* produces all four analogs, they are likely the product of the same biosynthetic pathway with the olefins installed at the same position. Therefore, we hypothesized that the sole olefin in **4** and **6** was most likely between C11 and C12. The observed loss of 85 Da in **3** and **5** is hypothesized to result from a radical rearrangement that shifts the olefin to the bicyclic system (Fig. S19).

**Scheme 2:**
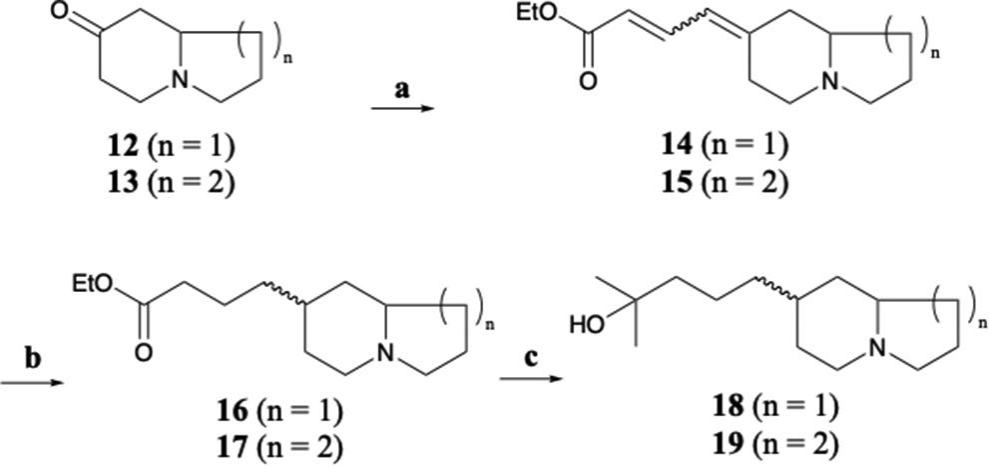
Synthesis of water adducts of **4** and **6**. (**a**) triethyl-4-phosphonocrotonate, THF, 0 ^o^C to rt, 16 h; (**b**) 10% Pd/C, EtOH, 3 atm, 45 min, rt; (**c**) MeMgCl, THF, 16 h, rt

To confirm these hypotheses, total syntheses were attempted to produce both the hydrate adducts of **4** and **6** from 8-indolizidone and 2-quinolizidone, respectively (Scheme 2). First, **12** and **13** were reacted with triethylphosphonocrotonate via a Horner-Wadsworth-Emmons reaction to form **14** and **15** (Fig. S20 and S21). Crude reaction mixtures were subsequently hydrogenated and treated with excess methyl magnesium chloride to yield hydroxy indolizidine (**18**) and hydroxy quinolizidine (**19**) (Fig. S22 - S23) (Namboothiri et al. 1997). Both product mixtures consisted of a 3:1 mixture of diastereomers, and the mass spectrum and retention time of the major (first eluting isomer) matched those of the hydrated natural alkaloids (Fig. S26 and SS27). Unfortunately, we were unable to separate the diastereomers using a range of LC methods and columns, so the products were analyzed by 2D NMR as mixtures (Fig. S28 - S39; Table S2 - S5). This led to complete assignments of both isomers for **18** and **19**, with the major isomer in both samples exhibiting a clear ROESY correlation between the two stereogenic methines, indicating a *syn* orientation. This was supported by the lack of a ROESY correlation between the same methines for the minor isomer (*anti* orientation).

In November 2023, we made a fortuitous collection of *B. producta* and this material was successfully used to isolate small quantities of both the hydro-analogs, **4** and **6**. Approximately 30 millipedes were extracted in methanol, and analysis of the crude extract by LCMS revealed both **4** and **6** as major components, while **3** and **5** were not present. Reverse-phase HPLC purification led to less than 0.5 mg of both **4** and **6**. Analysis of both compounds by 2D NMR confirmed their planar structures (Table S6 and S7, Fig. S40 – S50). In addition, a clear ROESY correlation was observed between H3 and H5 indicating a *syn* relationship between the methines. Assigning the absolute stereoconfiguration will require an enantiopure total synthesis of all four natural metabolites.

## Discussion

This investigation describes the chemistry of the defensive secretions from three previously unstudied species of *Brachycybe* millipedes, two of which are sister species (*B. petasata* and *B. producta*), and another closely related species (*B. rosea*). Although the sister species are non-sympatric**–***B. petasata* is found on the East Coast and *B. producta* is found on the West Coast of the U.S. **–**both produce the same bicyclic monoterpene alkaloids (**3–6**). Their secretions consist of two indolizidines [gosodesmine (**3**) and hydrogosodesmine (**4**)] and their quinolizidine homologs [homogosodesmine (**5**) and hydrohomogosodesmine (**6**)], marking the first time a quinolizidine-containing natural product has been isolated from a millipede. In addition, the presence of indolizidine and quinolizidine concomitants in the millipede secretions is reminiscent of the pumiliotoxins (indolizidines) and homopumiliotoxins (quinolizidines) that are found within the skin of poison dart frogs. Although originally discovered from studies of poison dart frogs, it has since been determined that the frogs acquire these defensive agents from their arthropod diet, particularly oribatid mites (Daly et al. 2003; Saporito et al. 2004, 2007). The pumiliotoxins are believed to serve as defensive metabolites as they exhibit potent toxicity against mice, with LD_50_ of 20 to 50 µg (Edwards et al. 1988; Saporito et al. 2004).

While the chemical identity of these alkaloids has been elucidated, their ecological function and the evolution of their biosynthesis remains unclear. The volume, concentration, and apparent effect on the millipedes’ predators, such as ants (rapid motor incapacitation), strongly implies a strong deterrent effect (Meinwald et al. 1966; Schildknecht et al. 1967; Carrel 1984). In support of a defensive role, many millipedes, including those producing alkaloids, secrete visible secretions from their ozopores (openings of the ozadenes, defense glands) when physically disturbed (Shorter et al. 2018). This includes the secretion of a white, milk-like substance when collected with forceps (field observations). In addition, there are a few examples of the alkaloids themselves deterring predation experimentally. For example, **1** has been shown to reduce the rate of attack by *Formica* ants when experimentally applied to mealworms, and polyzonimine produced by *Petaserpes rosalbus*, is known to act as an insect repellent at concentrations as low as 100 nM (Smolanoff et al. 1975; Wood et al. 2000). Thus, existing literature strongly supports a defensive role of the terpenoid alkaloid secretions. However, Shear (2015) raised the possibility that some of these compounds may also act as pheromones facilitating aggregation, as some members of the subterclass Colobognatha are social millipedes and are known to form aggregates on the undersides of decaying logs. Moreover, there are examples of high viscosity “sticky” components of secretions in Glomerida, Chordeumatida, and Siphonophorida (Carrell 1984, Marek et al. 2012; Meinwald et al. 1966; Schildknecht et al. 1966, Youngsteadt 2008). The functions of these are unclear, but functional hypotheses have been made such as clinging to stones, an antipredatory or antiparasite role, or a soil-shedding mechanism to allow efficient burrowing (Marek et al. 2012).

To date, sixteen terpenoid alkaloids have been isolated from ten genera of millipedes, all within the subterclass Colobognatha—spare Siphonophorida that produces monoterpenes (Shear 2015; Kunert et al. 2023). The chemical diversity of the alkaloids varies across the millipede phylogeny where the monoterpene alkaloids [**1–6**, and buzonamine] are produced by species of millipedes within two orders, Platydesmida and Polyzoniida, and spiro-alkaloids, such as polyzonimine, are found within Siphonocryptida and Polyzoniida. The buzonamine, indolizidine and quinolizidine monoterpene alkaloids appear to each consist of a monoterpene and pyrrolidine or piperidine but have undergone different cyclization steps. Further analysis of the monoterpene alkaloids, the bicyclic analogs (**3–6**) have only been isolated from two genera of millipedes, all belonging to Platydesmida, while the tricyclic monoterpenes have been isolated from genera representing both Platydesmida and Polyzoniida. Through this study, we have discovered that this tight relationship between phylogeny and chemical diversity holds true down to sister species. Despite *B. lecontii* and *B. petasata* both being known from the eastern U.S., even co-occurring on the same decaying logs at some sites, *B. petasata* is not the closest evolutionary sister of *B. lecontii*. Instead, the closest evolutionary sister of *B. lecontii* is *B. nodulosa*, endemic to Japan, and *B. rosea*, endemic to California. Based on a phylogeny of *Brachycybe* by Brewer et al. (2012), *B.petasata* is closely related to *Brachycybe producta*, a species from California (Brewer et al. 2012). Similarly, *B. producta* co-occurs with *Brachycybe rosea*, the latter which is more closely related to *B. lecontii* and *B. nodulosa*. The chemistry of their defensive secretions follows this evolutionary relationship, where *B. lecontii* and *B. rosea* produce the same tricyclic secretion (e.g. **1**), while *B. petasata* and *B. producta* both produce the same bicyclic secretions (e.g. **3–6**). This strong relationship between defensive gland secretion chemistry and phylogeny indicates that the millipedes are themselves producing the secretions *de novo* and suggests that the chemical diversity within the terpenoid alkaloids may arise from the evolution of cyclase genes.

The discovery of these new terpenoid alkaloids adds to the growing knowledge of millipede defensive secretions and is beginning to provide insights into how these compounds might be biosynthesized and their evolution within millipedes. However, additional chemical investigations into unstudied millipedes belonging to the subterclass Colobognatha are necessary to provide insights into key biosynthetic transformations and to fully understand the evolution of this interesting class of compounds. To date, eleven genera of Platydesmida, and 59 species have not yet been chemically investigated, and this cohort undoubtedly includes new terpenoid alkaloids yet to be discovered. This includes many related species, such as *B. nodulosa* from Japan and *B. picta* (sympatric to *B. producta)* from California, with the latter representing the earliest-diverging *Brachycybe* species that is evolutionary sister to others in the genus. Future analyses will undoubtedly provide additional insights into the evolution of these compounds.

## Electronic Supplementary Material

Below is the link to the electronic supplementary material.

Supplementary Material 1

## Data Availability

No datasets were generated or analysed during the current study.

## Declarations

### NIST Disclaimer

Certain equipment, instruments, software, or materials are identified in this paper in order to specify the experimental procedure adequately. Such identification is not intended to imply recommendation or endorsement of any product or service by NIST, nor is it intended to imply that the materials or equipment identified are necessarily the best available for the purpose.
