# Supplementary material for: The Chemistry of the Defensive Secretions of Three Species of Millipedes in the Genus Brachycybe
Source: J Chem Ecol. 2024 Jun 10;50(9-10):478–88. doi: 10.1007/s10886-024-01518-6 (PMC11493816; doi:10.1007/s10886-024-01518-6)
Supplement: Supplementary file 1 — Supplementary Material 1 [file 10886_2024_1518_MOESM1_ESM.docx]

SUPPORTING INFORMATION

THE CHEMISTRY OF THE DEFENSIVE SECRETIONS OF THREE SPECIES OF MILLIPEDES IN THE GENUS *Brachycybe*

PAIGE BANKS,^1^ EMMA M. FUNKHOUSER,^2^ ANGIE M. MACIAS,^3^ BRIAN LOVETT,^4^ SHELBY MEADOR,^3^ ARDEN HATCH,^1^ H. MARTIN GARRAFFO,^5,6^ KAITIE C. CARTWRIGHT,^2^ PAUL MAREK,^7^ MATT T. KASSON,^3^ TAPPEY H. JONES,^2^ EMILY MEVERS^1^

Addresses

*^1^Department of Chemistry, Virginia Tech, Blacksburg, VA 24060, ^2^Department of Chemistry, Virginia Military Institute, Lexington, VA 24450, ^3^Division of Plant and Soil Sciences, West Virginia University, Morgantown, WV 26506, ^4^Emerging Pests and Pathogens Research Unit, USDA ARS, Ithaca, NY 14853, ^5^National Institute of Diabetes and Digestive and Kidney Diseases, National Institutes of Health, Bethesda, MD 20892, ^6^National Institute of Standards and Technology, Gaithersburg, MD 20899, ^7^Department of Entomology, Virginia Tech, Blacksburg, VA 24060*

**Table of Contents**

**Figure S1.** GCMS chromatogram and spectra of a representative *B. petaseta* extract……………......pg. 4

**Figure S2.** GCMS chromatogram and spectra of a representative *B. producta* extract……...………..pg. 5

**Figure S3.** GCMS chromatogram and spectrum of a representative *B. rosea* extract...……………....pg. 6

**Figure S4.** GCMS chromatogram and spectrum of a representative *B. lecontii* extract………...…….pg. 7

**Figure S5.** GCMS chromatogram and spectra of the hydrogenation reaction product from *B. petasata*………………………………………………………………………………………………...pg. 7

**Figure S6.** GCMS chromatogram and spectra of the hydrogenation reaction product from *B. producta*…………………………………………………………………………………………......…pg. 8

**Figure S7.** GCMS chromatogram and spectrum of the synthesized **7**……………...………...………pg. 9

**Figure S8.** GCMS chromatogram and spectrum of the synthesized **8**……………...………...………pg. 9

**Figure S9.** GCMS chromatogram and spectrum of the synthesized **5**……………...………….……..pg. 10

**Figure S10.** GCMS chromatogram and spectrum of the synthesized **11**…………...………….……..pg. 11

**Figure S11.** Numbering of compounds for NMR data tables…………………...……………………pg. 12

**Table S1.** NMR Spectroscopy Data for synthetic homo-gosodesmine (**5**)...........................................pg. 12

**Figure S12.** ^1^H NMR spectrum for synthetic homogosodesmine (**5**)...................................................pg. 13

**Figure S13.** gHSQC for synthetic homogosodesmine (**5**).....................................................................pg. 13

**Figure S14.** H2BC for synthetic homogosodesmine (**5**).......................................................................pg. 14

**Figure S15.** HMBC for synthetic homogosodesmine (**5**)......................................................................pg. 14

**Figure S16.** dqfCOSY for synthetic homogosodesmine (**5**).................................................................pg. 15

**Figure S17.** Hydration product from reaction of *B. petesata* crude extract…………………………..pg. 16

**Figure S18.** Fragmentation of **18** and **19**……………………………………………………………...pg. 16

**Figure S19.** Plausible fragmentation mechanism for both **4** and **6**…………………………………...pg. 16

**Figure S20.** GCMS chromatogram and spectrum of the synthesized **14**…………....………………..pg. 17

**Figure S21.** GCMS chromatogram and spectrum of the synthesized **15**……………………..……....pg. 18

**Figure S22.** GCMS chromatogram and spectrum of the synthesized **16**………....…………….…….pg. 19

**Figure S23.** GCMS chromatogram and spectrum of the synthesized **17**…….…...…………..………pg. 20

**Figure S24.** GCMS chromatogram and spectrum of the synthesized **18**….……...………………..…pg. 21

**Figure S25.** GCMS chromatogram and spectrum of the synthesized **19**….……...………………..…pg. 22

**Figure S26.** Comparison of synthetic **18** with hydratation of *B. petasata* extract.……………….…..pg. 23

**Figure S27.** Comparison of synthetic **19** with hydratation of *B. petasata* extract……………………pg. 24

**Table S2.** NMR Spectroscopy Data for the synthetic *syn-*isomer of **18**………………………………pg. 25

**Table S3.** NMR Spectroscopy Data for the synthetic *anti-*isomer of **18**……………………………...pg. 26

**Figure S28.** ^1^H NMR spectrum for compound **18**………….…………………………………………pg. 27

**Figure S29.** gHSQC for compound **18**………………………………………………………………..pg. 27

**Figure S30.** H2BC for compound **18**…………………………………………...…………………….pg. 28

**Figure S31.** HMBC for compound **18**………………………………………………………………...pg. 28

**Figure S32.** dqfCOSY for compound **18** ……………………………………………………………..pg. 29

**Figure S33.** easyROESY for compound **18**…………………………………...……………………...pg. 29

**Table S4.** NMR Spectroscopy Data for the synthetic *syn-*isomer of compound **19**…………………..pg. 30

**Table S5.** NMR Spectroscopy Data for the synthetic *anti-*isomer of compound **19**………………….pg. 31

**Figure S34.** ^1^H NMR spectrum for compound **19**………….…………………………………………pg. 32

**Figure S35.** gHSQC for compound **19**………………………………………………………………..pg. 32

**Figure S36.** H2BC for compound **19**……………………………...…………………………….……pg. 33

**Figure S37.** HMBC for compound **19**………………………………………………………………...pg. 33

**Figure S38.** dqfCOSY for compound **19**………………………………………………………….…..pg. 34

**Figure S39.** easyROESY for compound **19**……………………...……………………………….…..pg. 34

**Table S6.** NMR Spectroscopy Data for natural hydrogosodesmine (**4**).……………………………...pg. 35

**Figure S40**. ^1^H NMR spectrum for hydrogosodesmine (**4**)……….…………………………………..pg. 35

**Figure S41**. gHSQC for hydrogosodesmine (**4**)………………………………………………………pg. 36

**Figure S42.** H2BC for hydrogosodesmine (**4**) …………………………………...…………………..pg. 36

**Figure S43.** HMBC for hydrogosodesmine (**4**)……………………………………………………….pg. 37

**Figure S44.** easyROESY for hydrogosodesmine (**4**) …………………………...……………………pg. 37

**Table S7.** NMR Spectroscopy Data for natural homo-hydrogosodesmine (**6**)...……………………..pg. 38

**Figure S45**. ^1^H NMR spectrum for homo-hydrogosodesmine (**6**) …………………………………...pg. 38

**Figure S46.** gHSQC for homo-hydrogosodesmine (**6**)………………………...……………………..pg. 39

**Figure S47.** H2BC for homo-hydrogosodesmine (**6**)………………………………………………...pg. 39

**Figure S48.** HMBC for homo-hydrogosodesmine (**6**)………………………...……………………...pg. 40

**Figure S49.** dqfCOSY for homo-hydrogosodesmine (**6**)……………………………………………..pg. 40

**Figure S50.** easyROESY for homo-hydrogosodesmine (**6**)…………………………………………..pg. 41

**
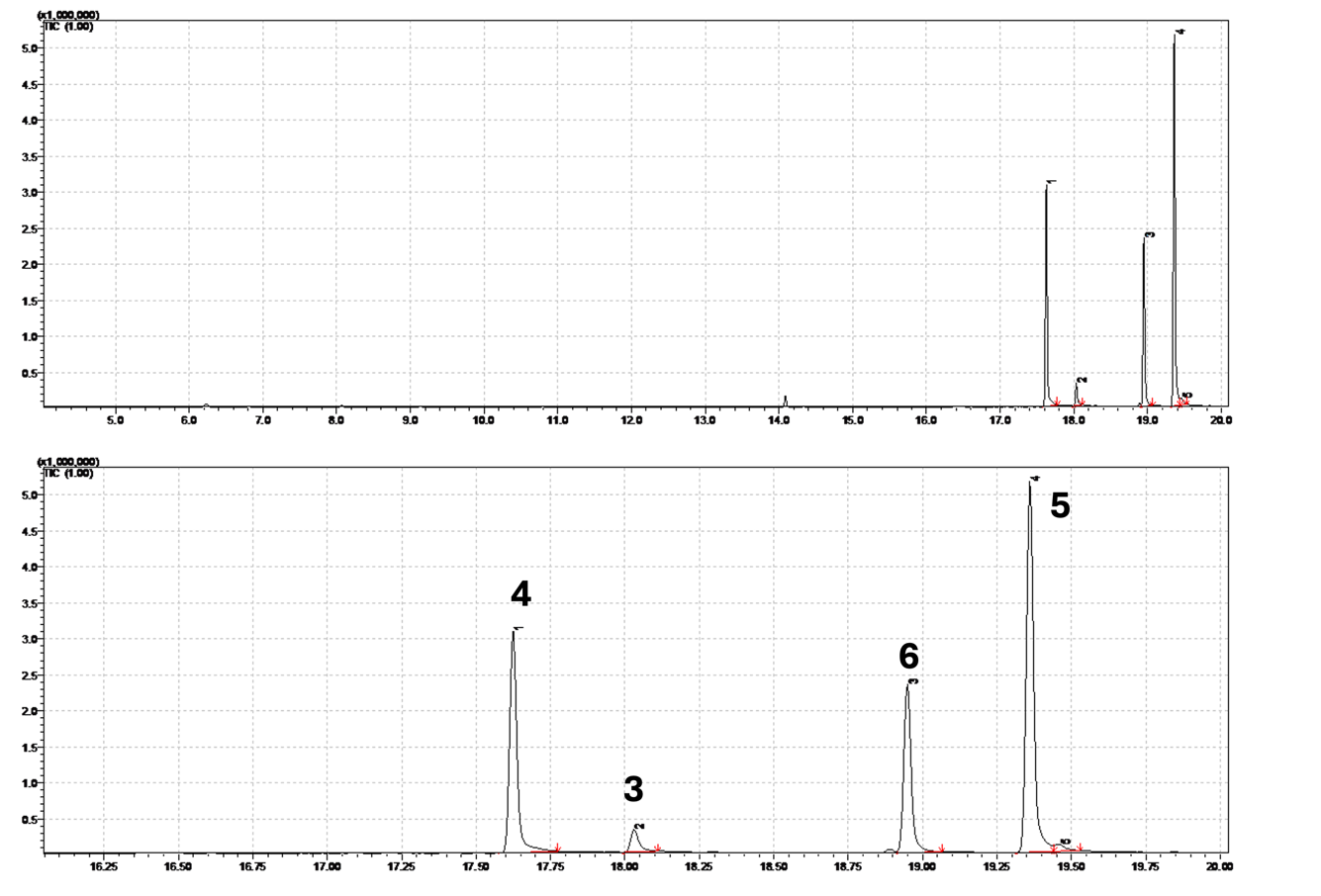
**

**
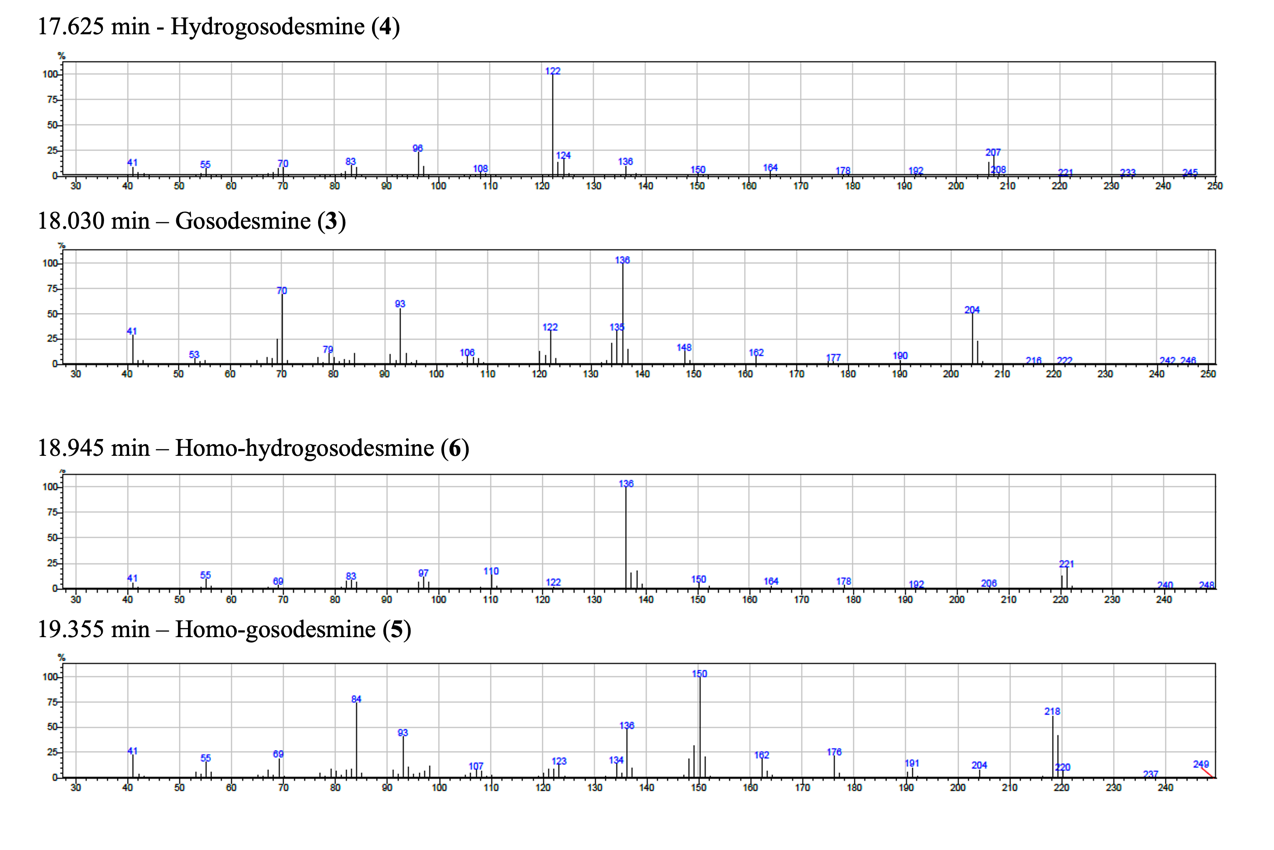
**

**Figure S1.** GCMS chromatogram and spectra of a representative *B. petaseta* extract (PEM-2021-008). Peaks at 6.3 min is α-pinene and 14 min is borneyl acetate.

**
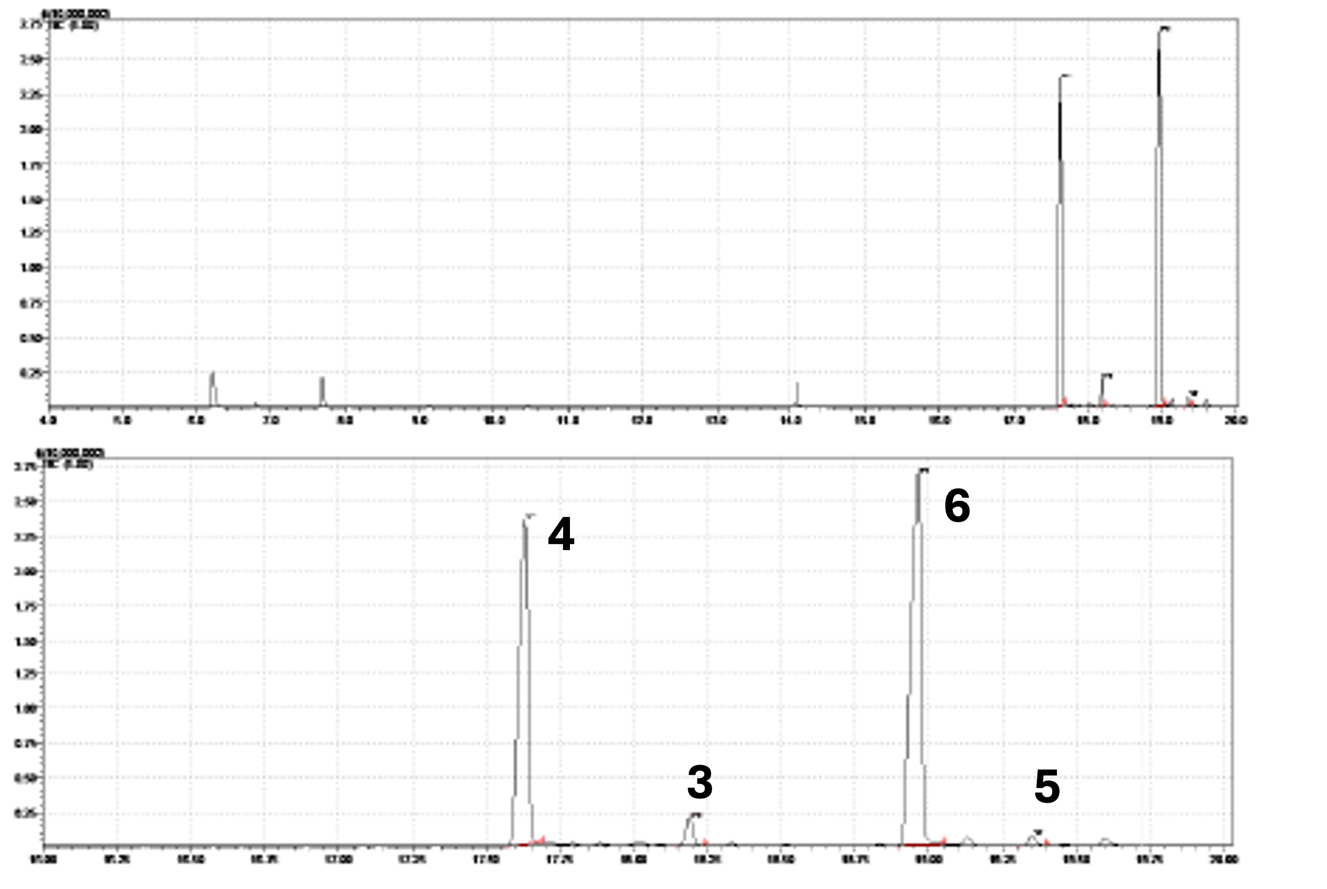
**


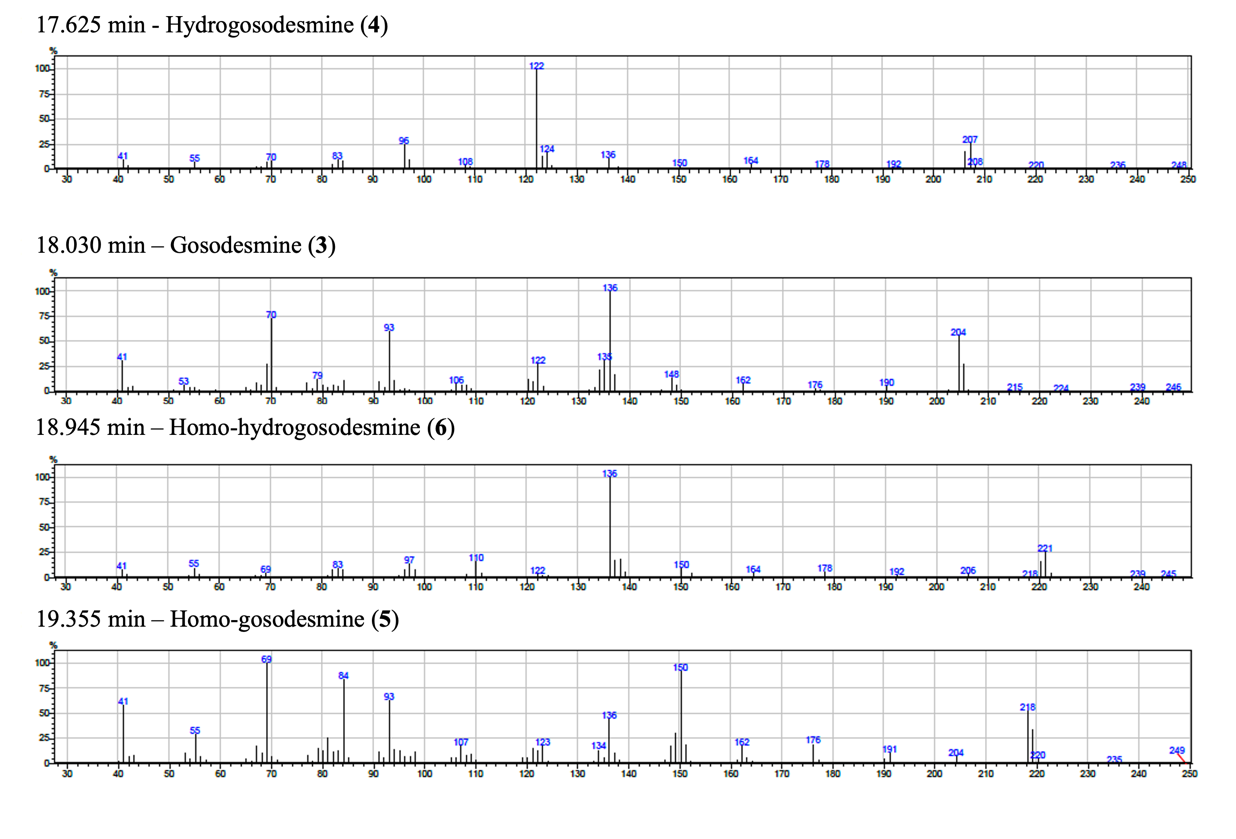


**Figure S2.** GCMS chromatogram and spectra of a representative *B. producta* extract

**
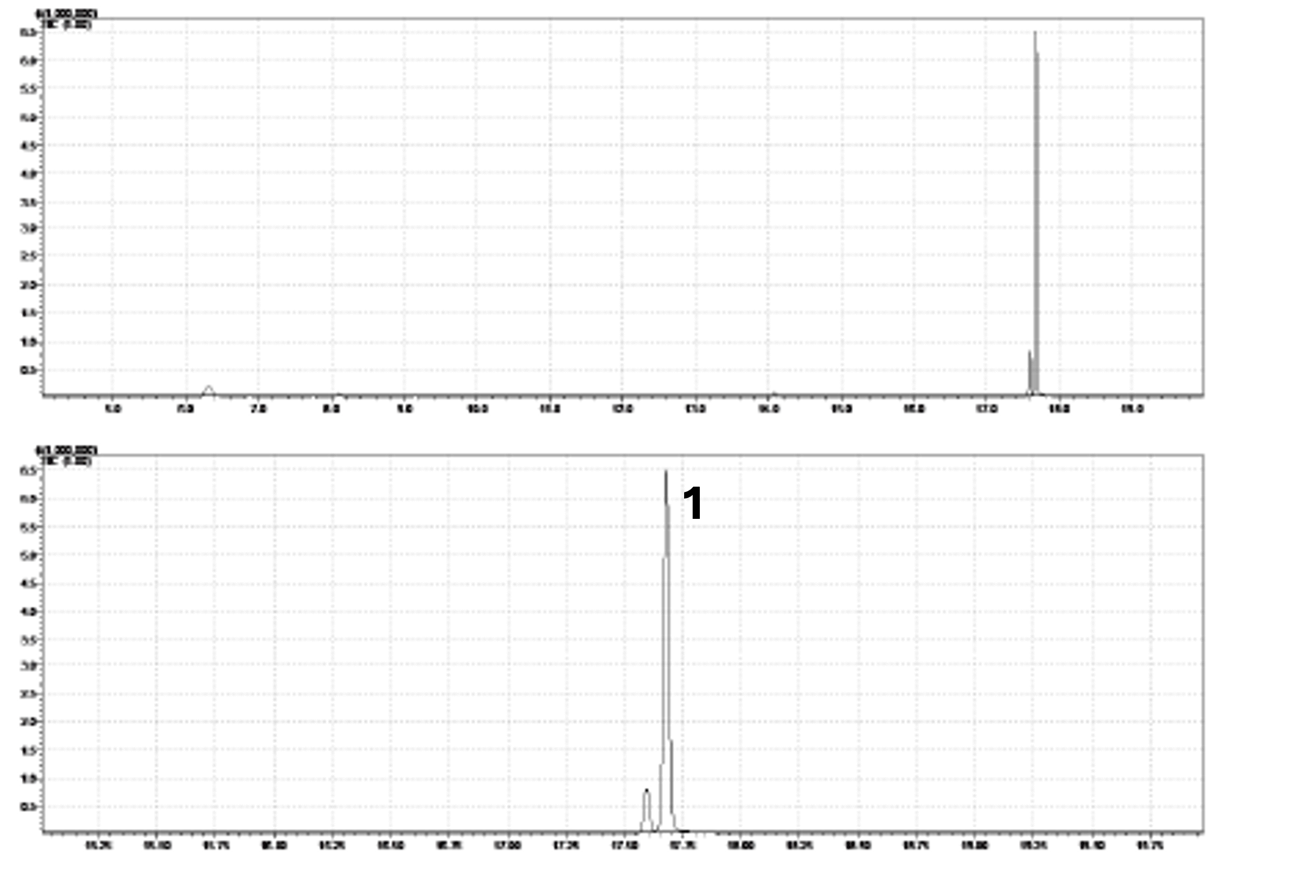

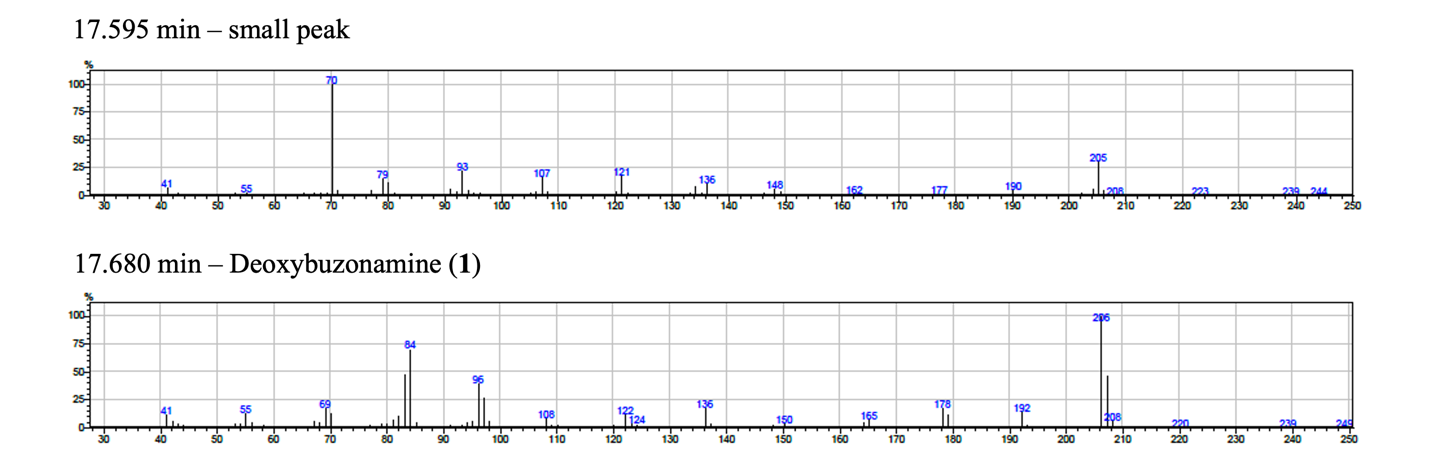
**

**Figure S3.** GCMS chromatogram and spectra of a representative *B. rosea* extract. The smaller peak at 17.595 min appears related to deoxybuzonamine. The fragment at m/z = 70 which suggests a of an olefin the middle ring but we did not have enough material for structure elucidation.


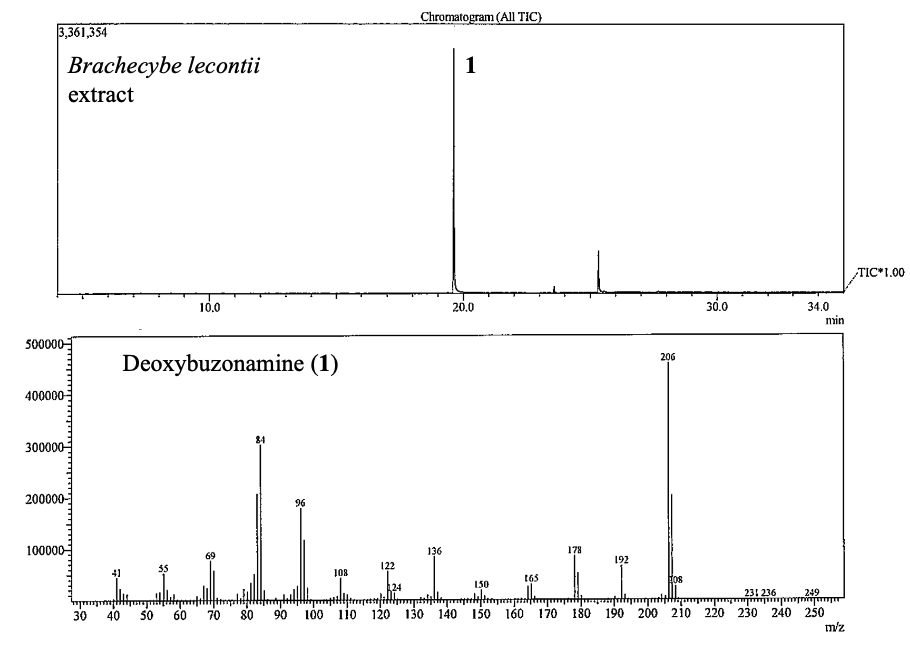


**Figure S4.** GCMS chromatogram and spectrum of a representative *B. lecontii* extract

**
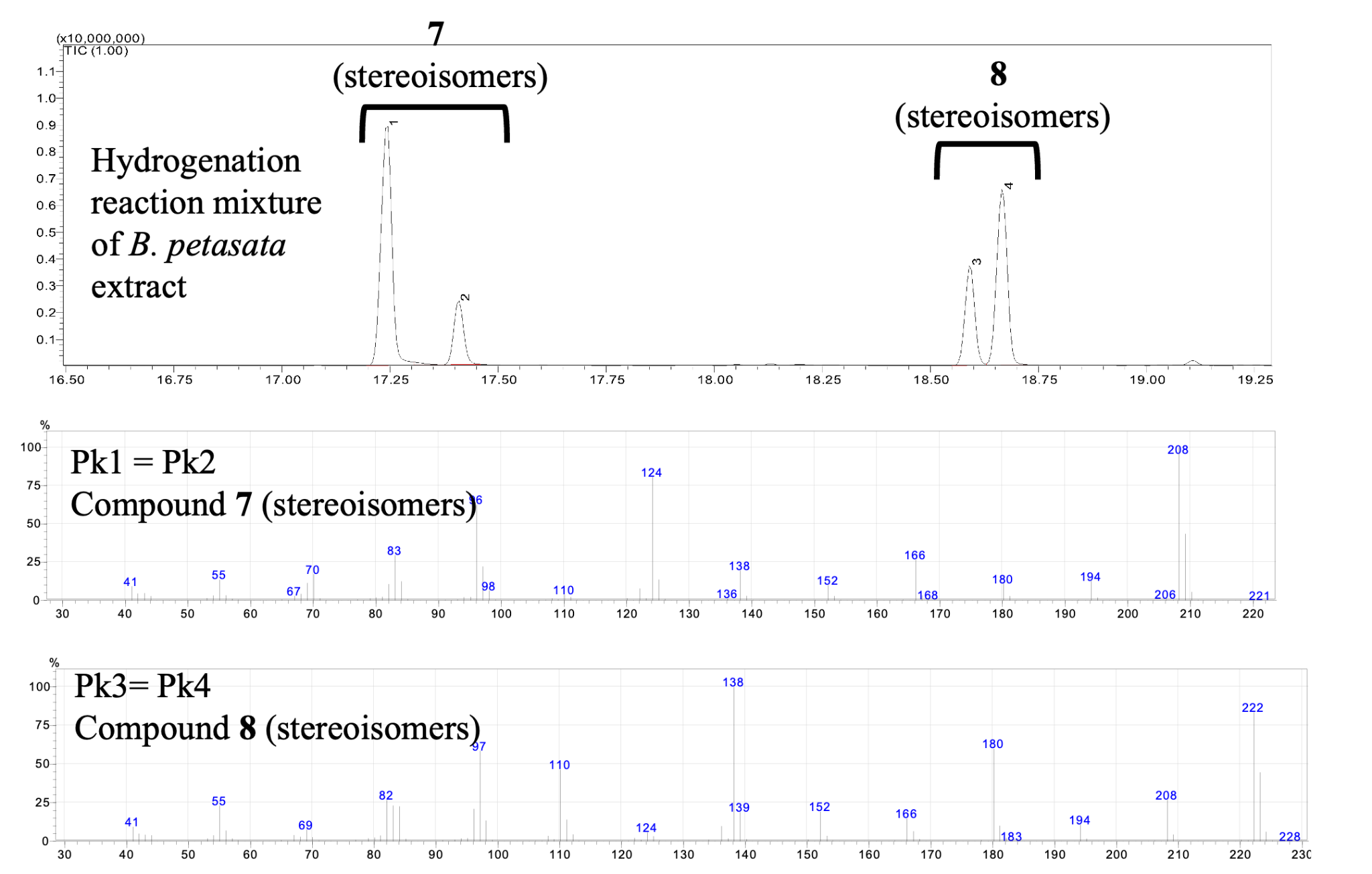
**

**Figure S5.** GCMS chromatogram and spectra of the reaction product of the hydrogenation of *B. petasata* extract. The reduction of gosodesmine (**3**) and homogosodesmine (**5)** led to stereoisomers. The fragmentation patterns of the stereoisomers were identical.

**
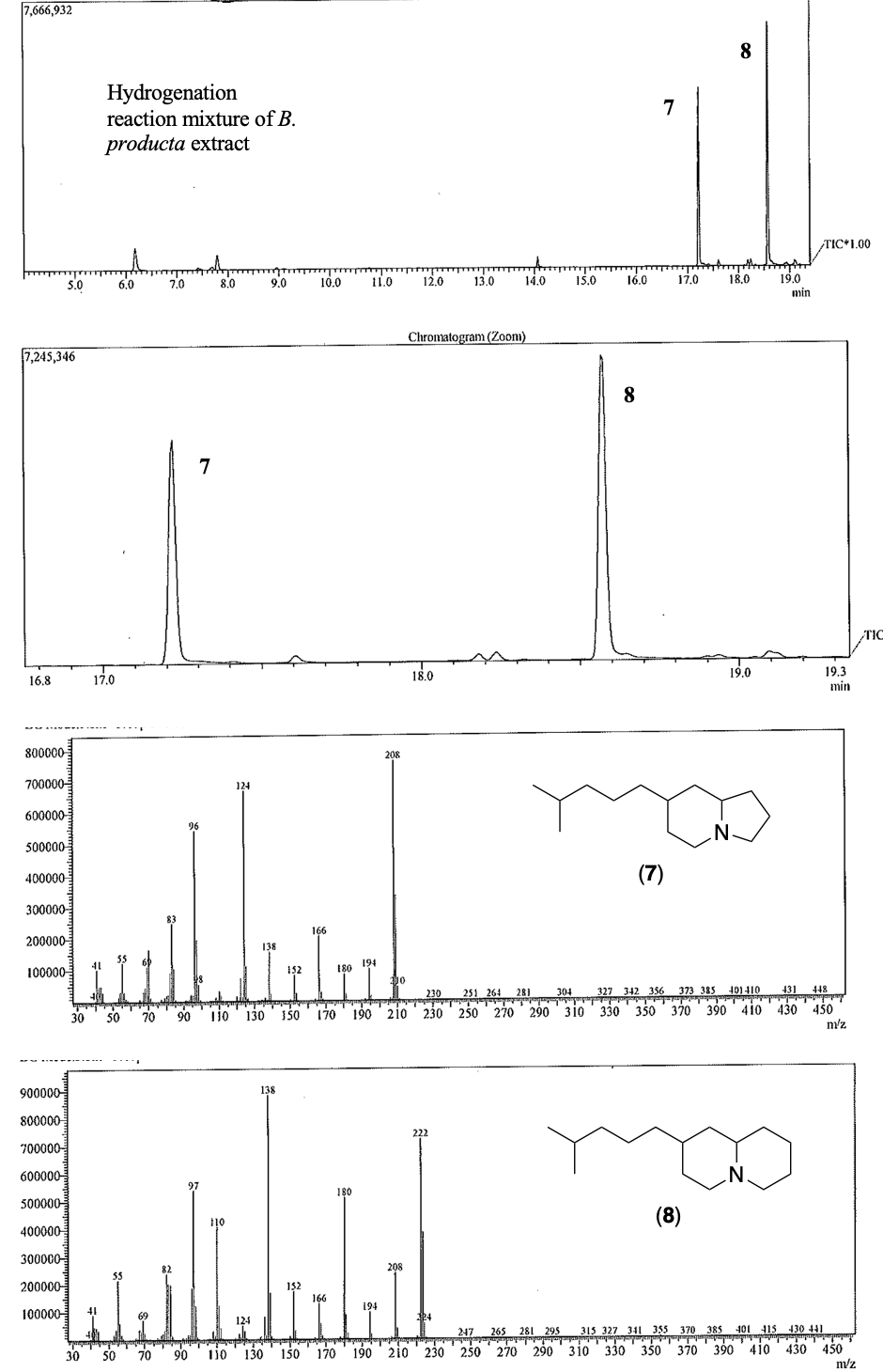
**

**Figure S6.** GCMS chromatogram and spectra of the reaction product of the hydrogenation of *B. producta* extract. The reduction of gosodesmine (**3**) and homogosodesmine (**5)** led to stereoisomers. The fragmentation patterns of the stereoisomers were identical.

**
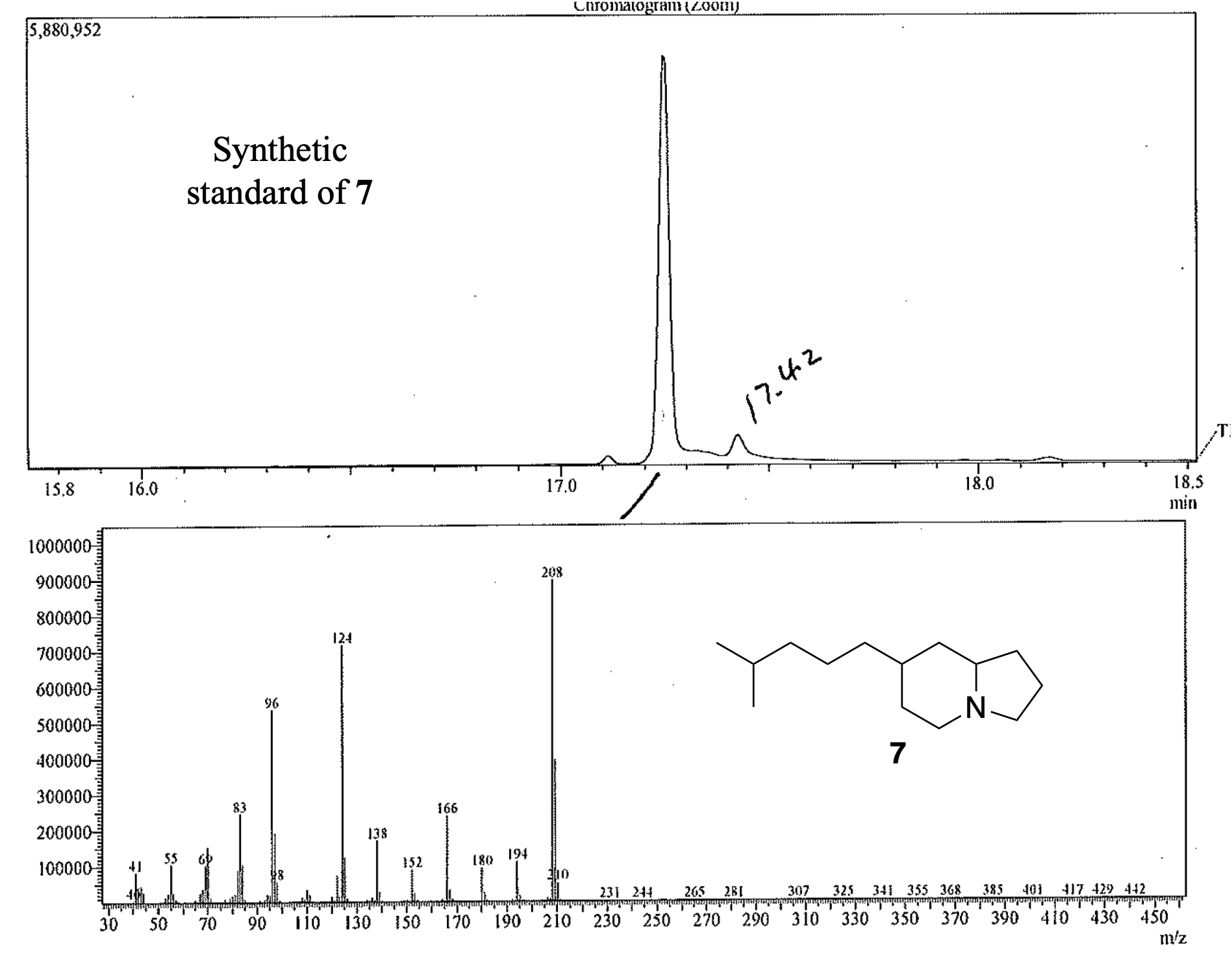
**

**Figure S7.** GCMS chromatogram and spectrum of the synthesized 7-(4-methylpentyl)-indolizidine (**7**)

**
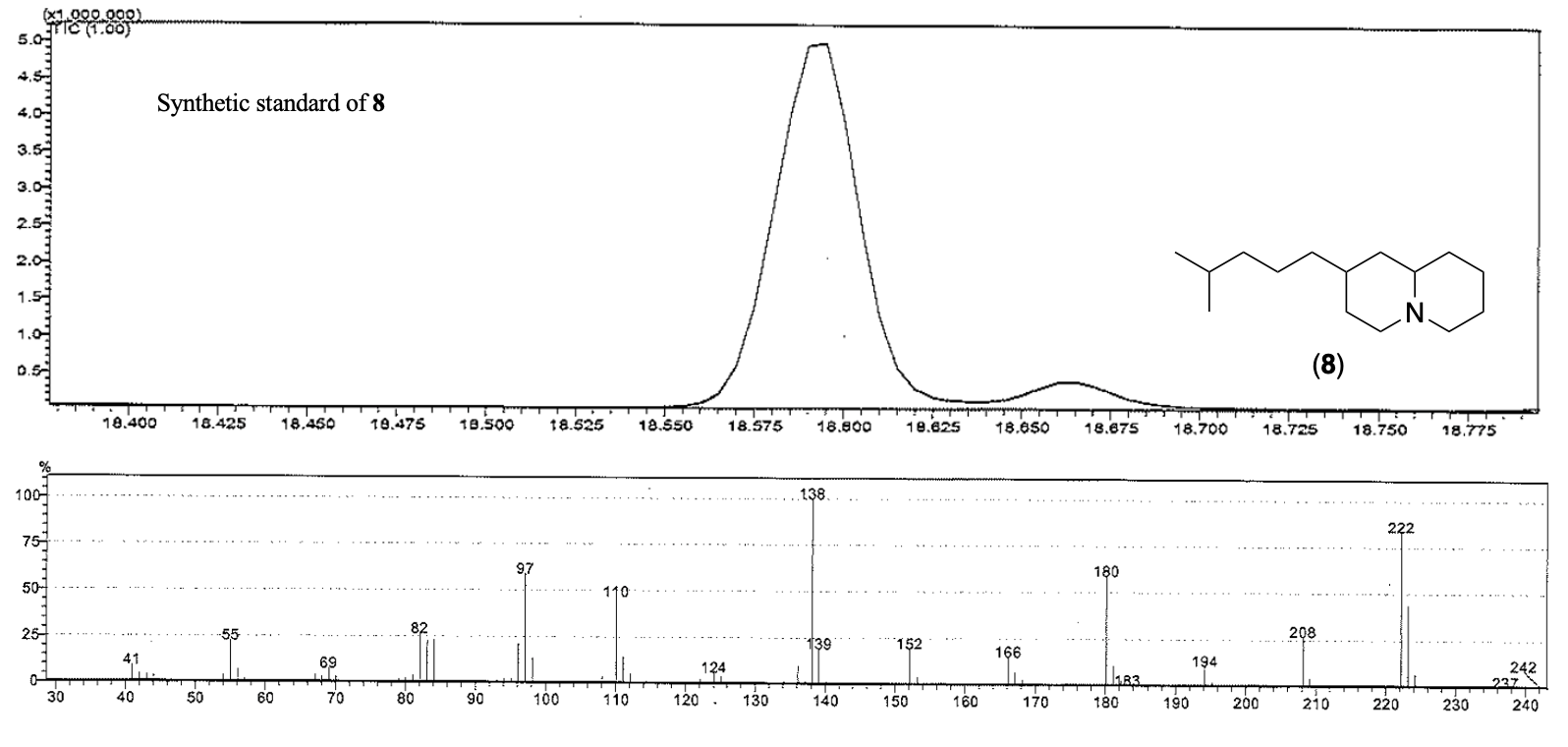
**

**Figure S8.** GCMS chromatogram and spectrum of the synthesized 2-(4-methylpentyl)-quinolizidine (**8**)


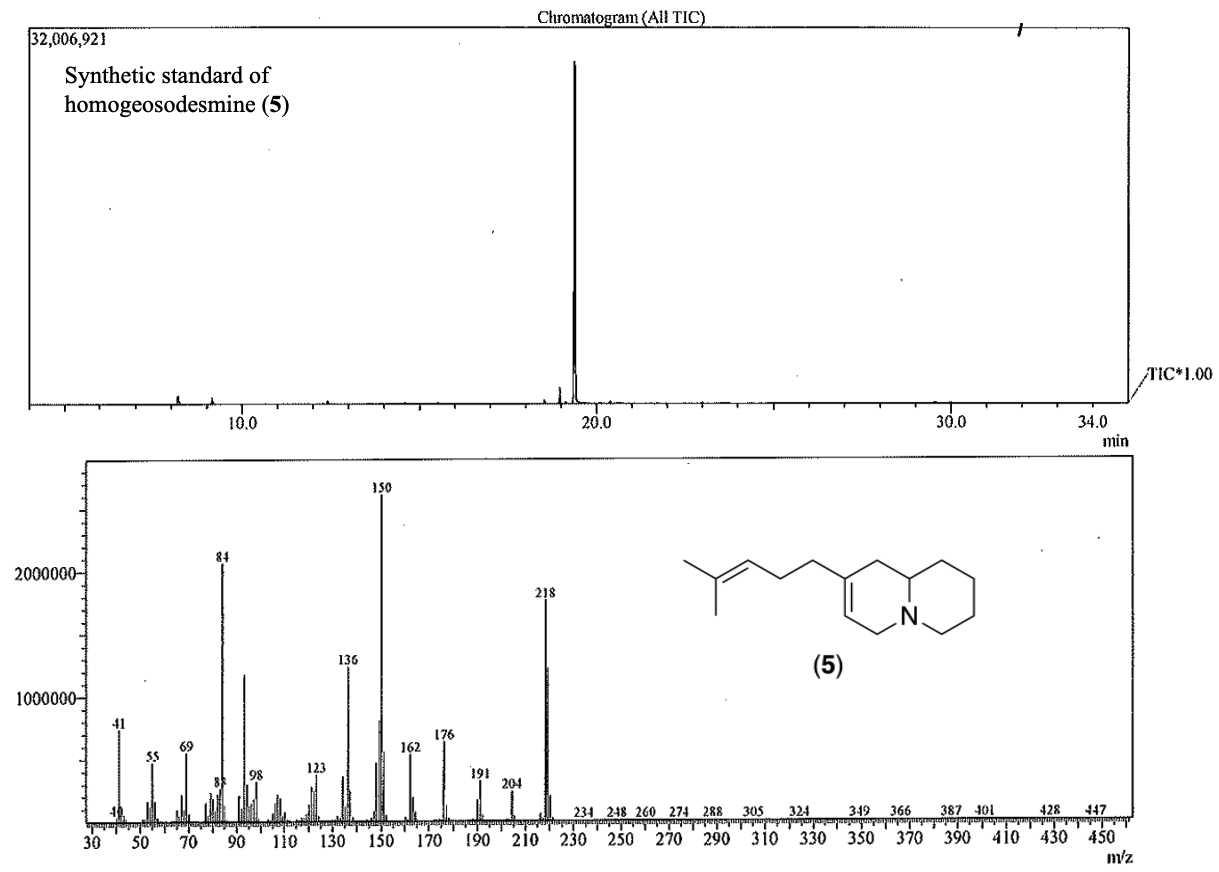


**Figure S9.** GCMS chromatogram and spectrum of the synthesized homogosodesmine (**5**)


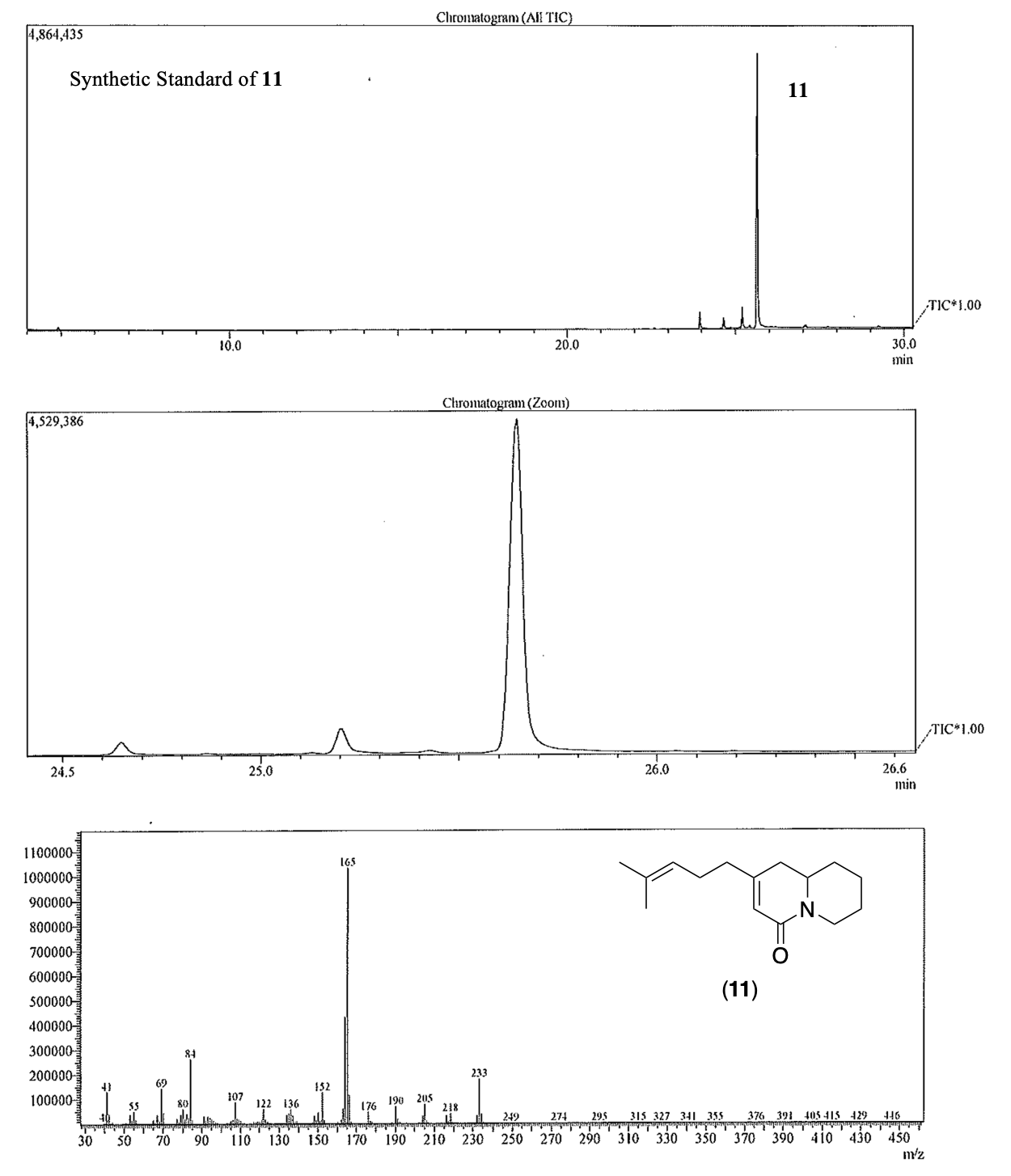
**Figure S10.** GCMS chromatogram and spectrum of the synthesized **11**


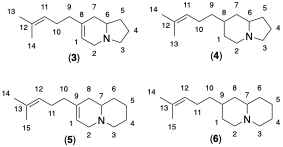


**Figure S11.** Numbering of compounds for NMR data tables

| **Table S1.** NMR Spectroscopy Data [600 MHz, (CD_3_)_2_SO, 50^o^C] for synthetic homo-gosodesmine (**5**) | | | | | |
| --- | --- | --- | --- | --- | --- |
| **Position** | **δ_C_, type^[a]^** | **δ_H_ (J in Hz)** | **H2BC** | **HMBC** | **COSY** |
| 1 | 124.4, CH | 5.09, m |  |  | 8 |
| 2 | 61.3, CH_2_ | 4.02, m |  |  | 8 |
| 3 | 54.0, CH_2_ | 4.51, m |  |  | 4 |
| 4 | 20.4, CH_2_ | 2.02, m | 4, 5 |  | 3, 5 |
| 5 | 16.8, CH_2_ | 1.88, m |  |  | 4, 6 |
| 6 | 27.3, CH_2_ | 3.16, m | 5 | 4, 5 | 5 |
| 7 | ND^[b]^ |  |  |  |  |
| 8 | 28.6, CH_2_ | 1.24, m |  |  | 1, 2 |
| 9 | 132.5, C |  |  |  |  |
| 10 | 34.3, CH_2_ | 2.84, t (7.62) |  | 12 | 11 |
| 11 | 27.1, CH_2_ | 2.36, m | 10 |  | 10, 12 |
| 12 | 122.0, CH | 5.11, m |  |  | 11 |
| 13 | 130.3, C |  |  |  |  |
| 14 | 17.3, CH_3_ | 1.56, m |  | 12, 13, 15 |  |
| 15 | 25.1, CH_3_ | 1.64, m |  | 12, 13, 14 |  |

^[a]^δ_C_ obtained indirectly from gHSQC and gHMBC experiments. ^[b]^ND: Not detected. Significant peak broadening occurred in signals near the tertiary amine. This led to weak and/or missing signals.

**
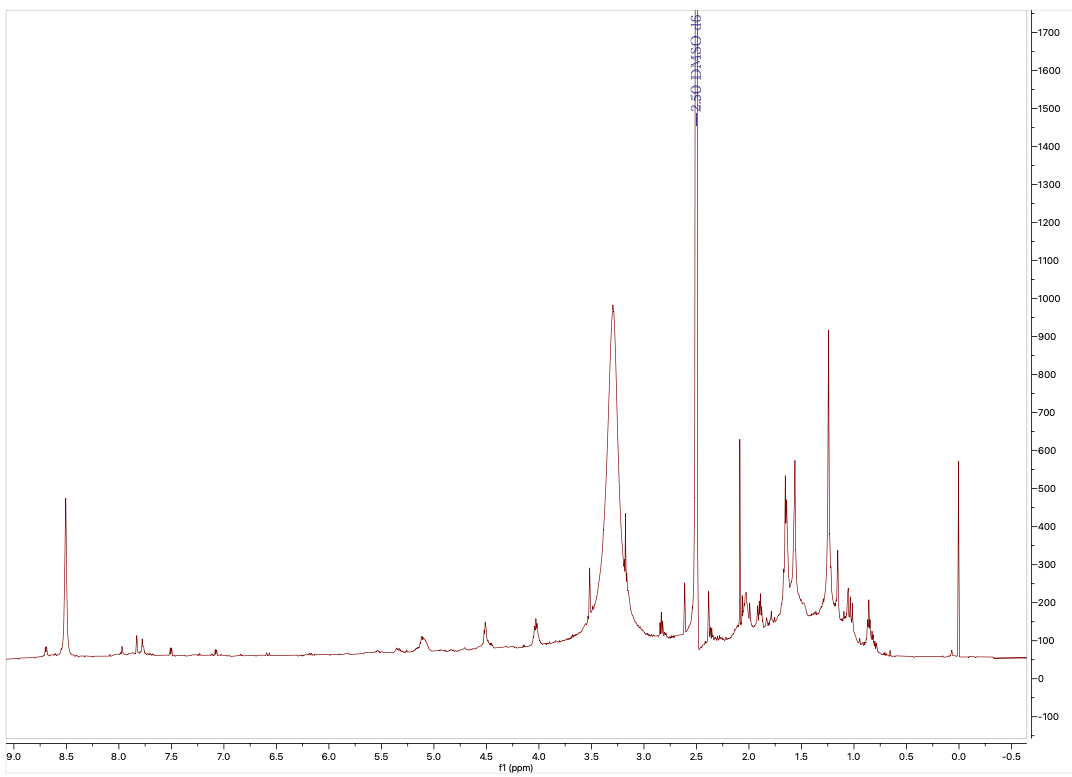
**

**Figure S12.** ^1^H NMR spectrum for synthetic homogosodesmine (**5**) (600 MHz, *d_6_*-DMSO). 128 scans


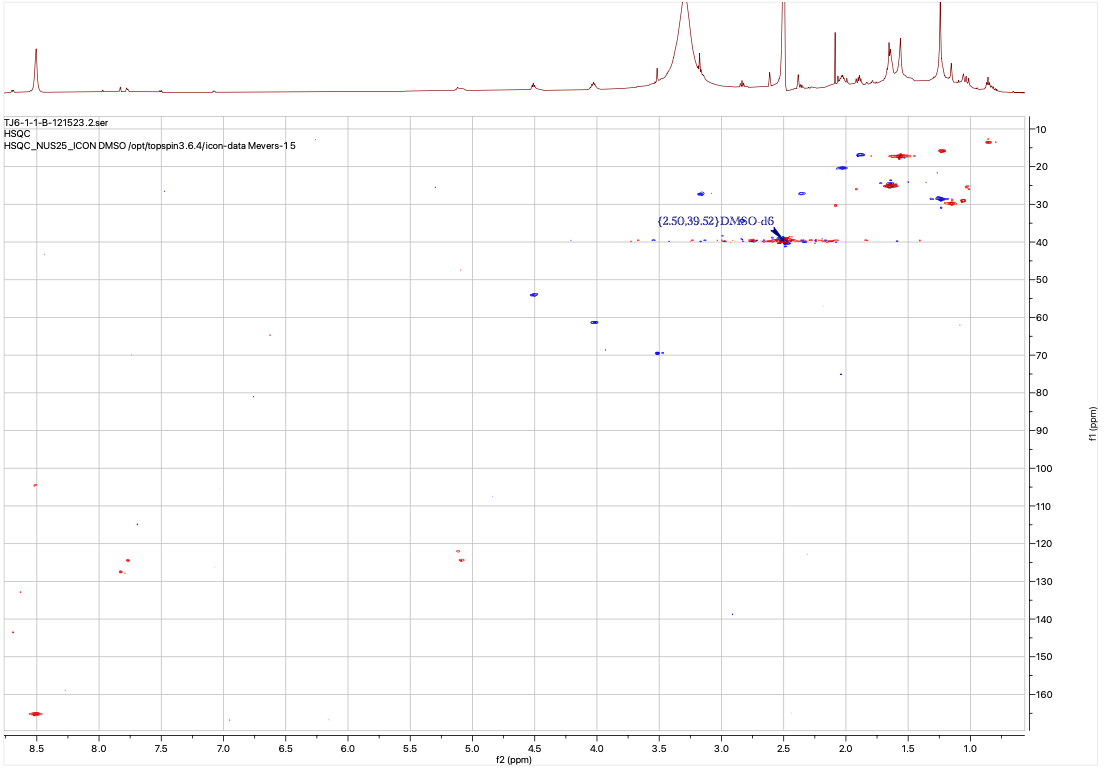


**Figure S13.** gHSQC for synthetic homogosodesmine (**5**) (600 MHz, *d_6_*-DMSO). 96 scans, NUS25 and 400 increments


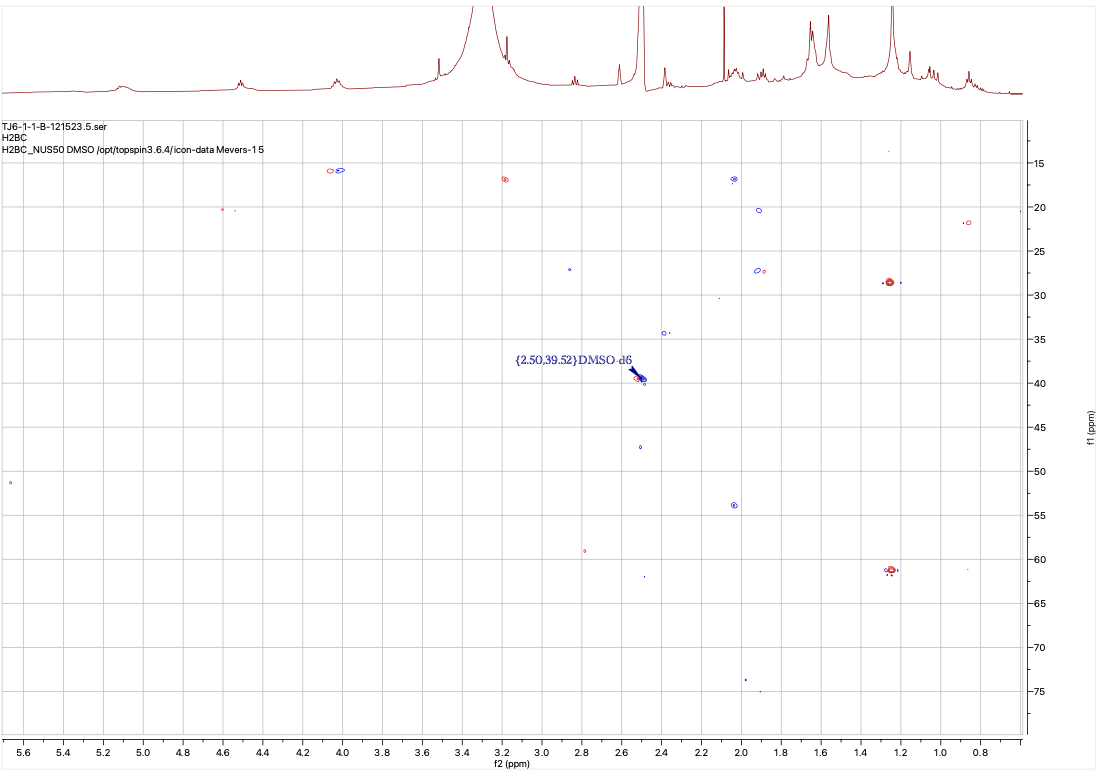


**Figure S14.** H2BC for synthetic homogosodesmine (**5**) (600 MHz, *d_6_*-DMSO). 160 scans, NUS50 and 400 increments.


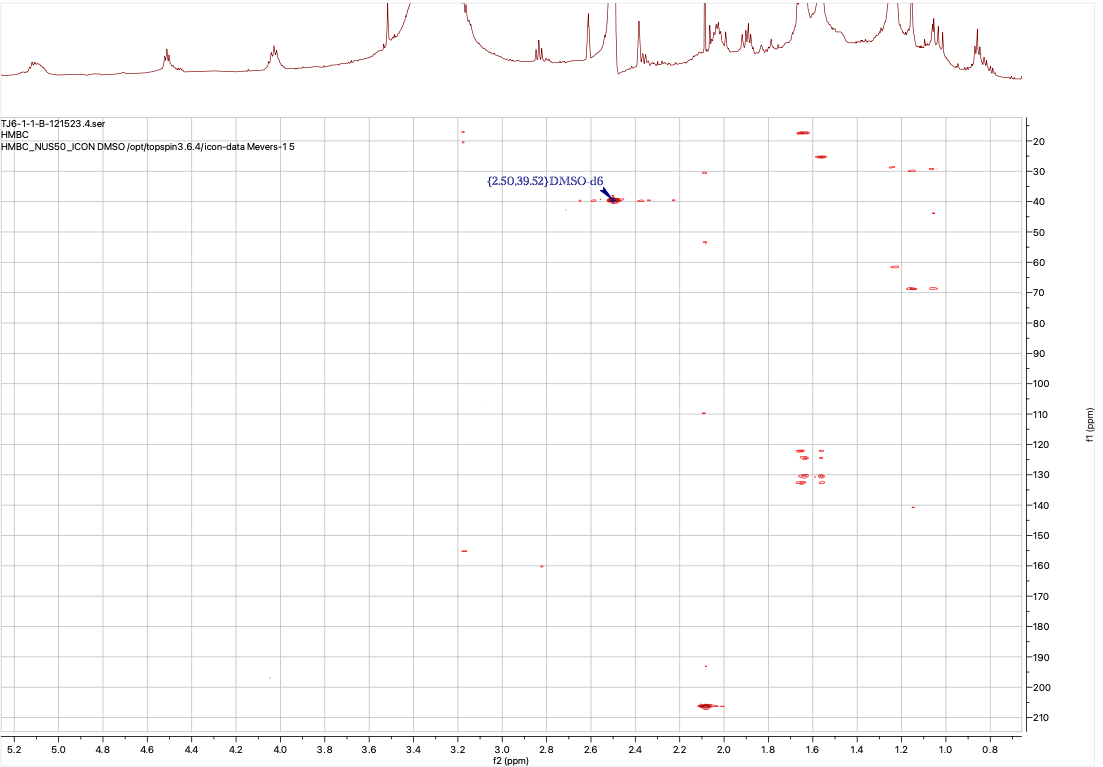


**Figure S15.** HMBC for synthetic homogosodesmine (**5**) (600 MHz, *d_6_*-DMSO). 144 scans, NUS50 and 512 increments.


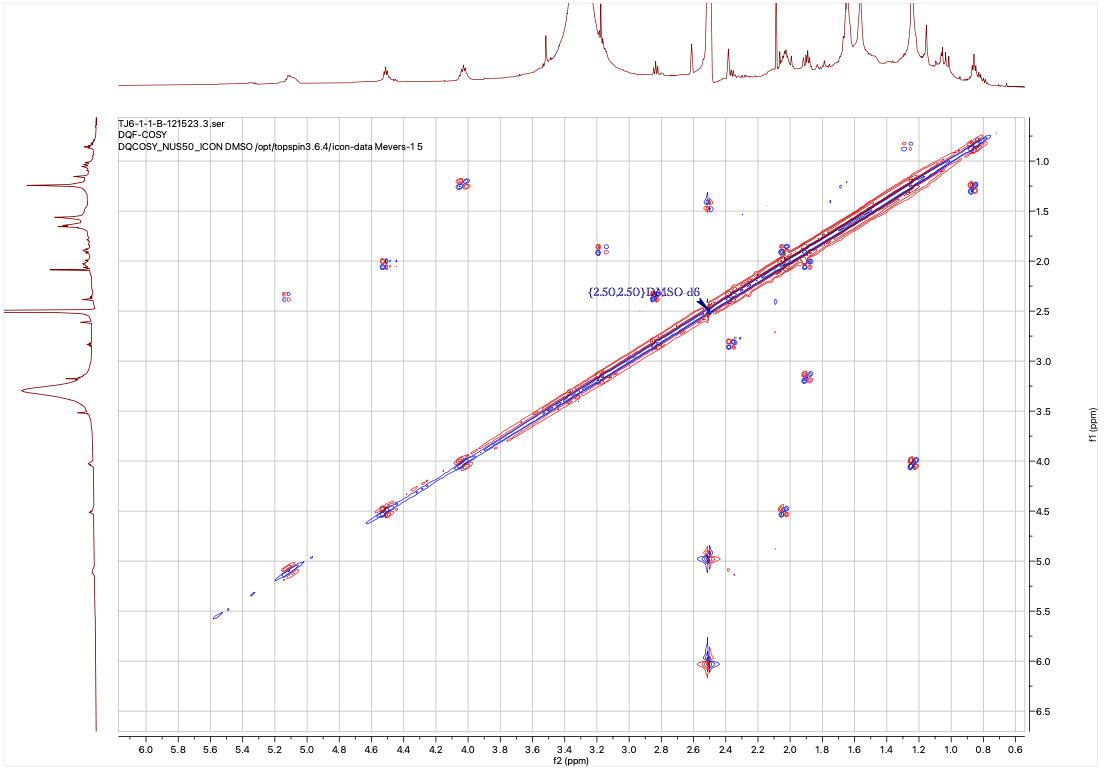


**Figure S16.** dqfCOSY for synthetic homogosodesmine (**5**) (600 MHz, *d_6_*-DMSO). 96 scans, NUS50 and 400 increments.


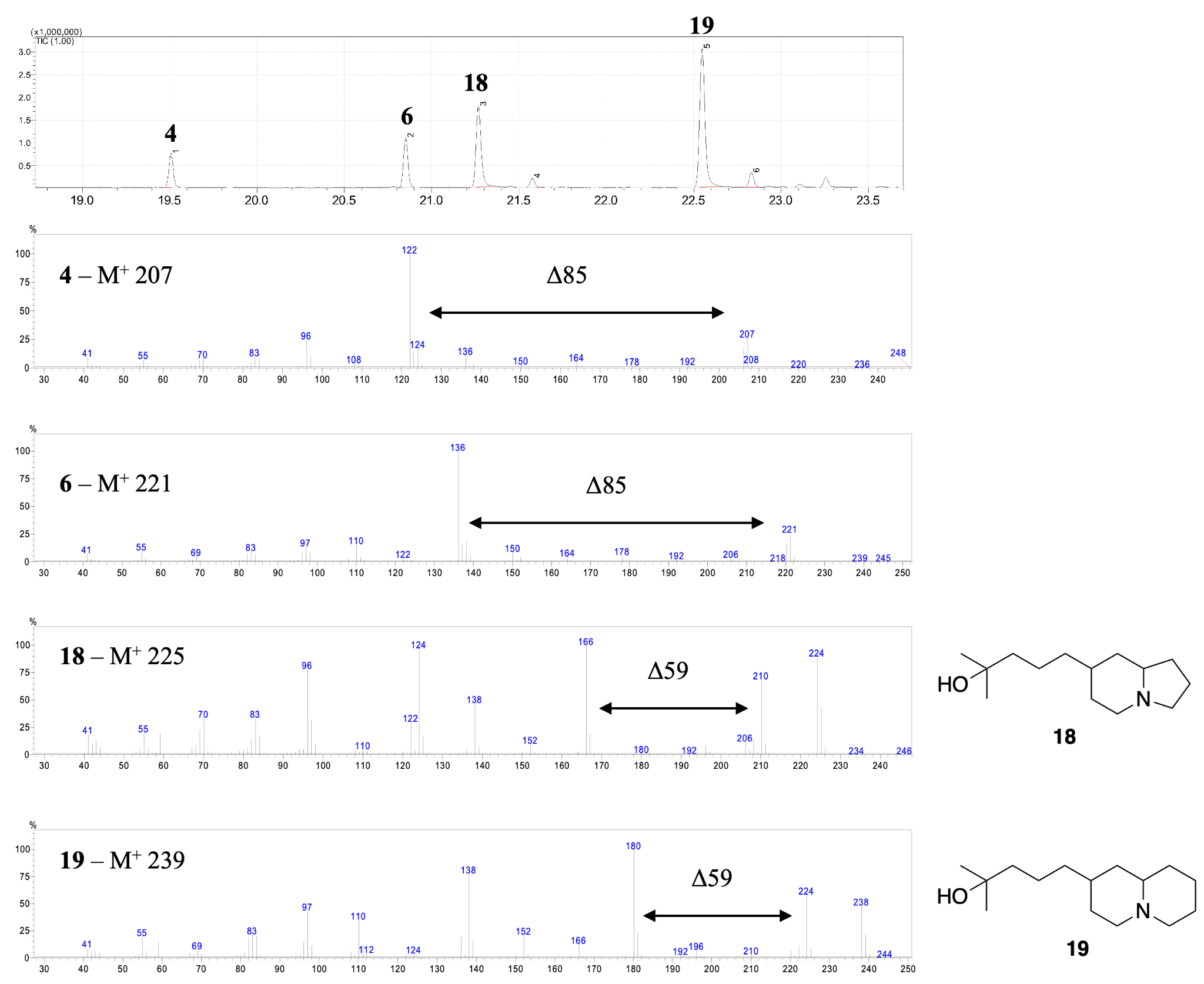


**Figure S17.** Hydration product from reaction of *B. petesata* crude extract with dilute HCl (aq). Compound **4** converted to **18** and **6** converted to **19**.


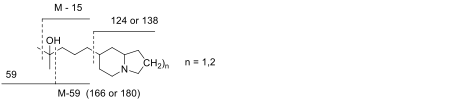


**Figure S18.** Fragmentation of **18** and **19**, the hydrate adducts of **4** and **6**, respectively.


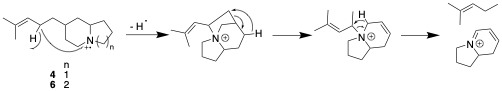


**Figure S19.** Plausible fragmentation mechanism that leads to observed loss of 85 Da in both **4** and **6**.

**
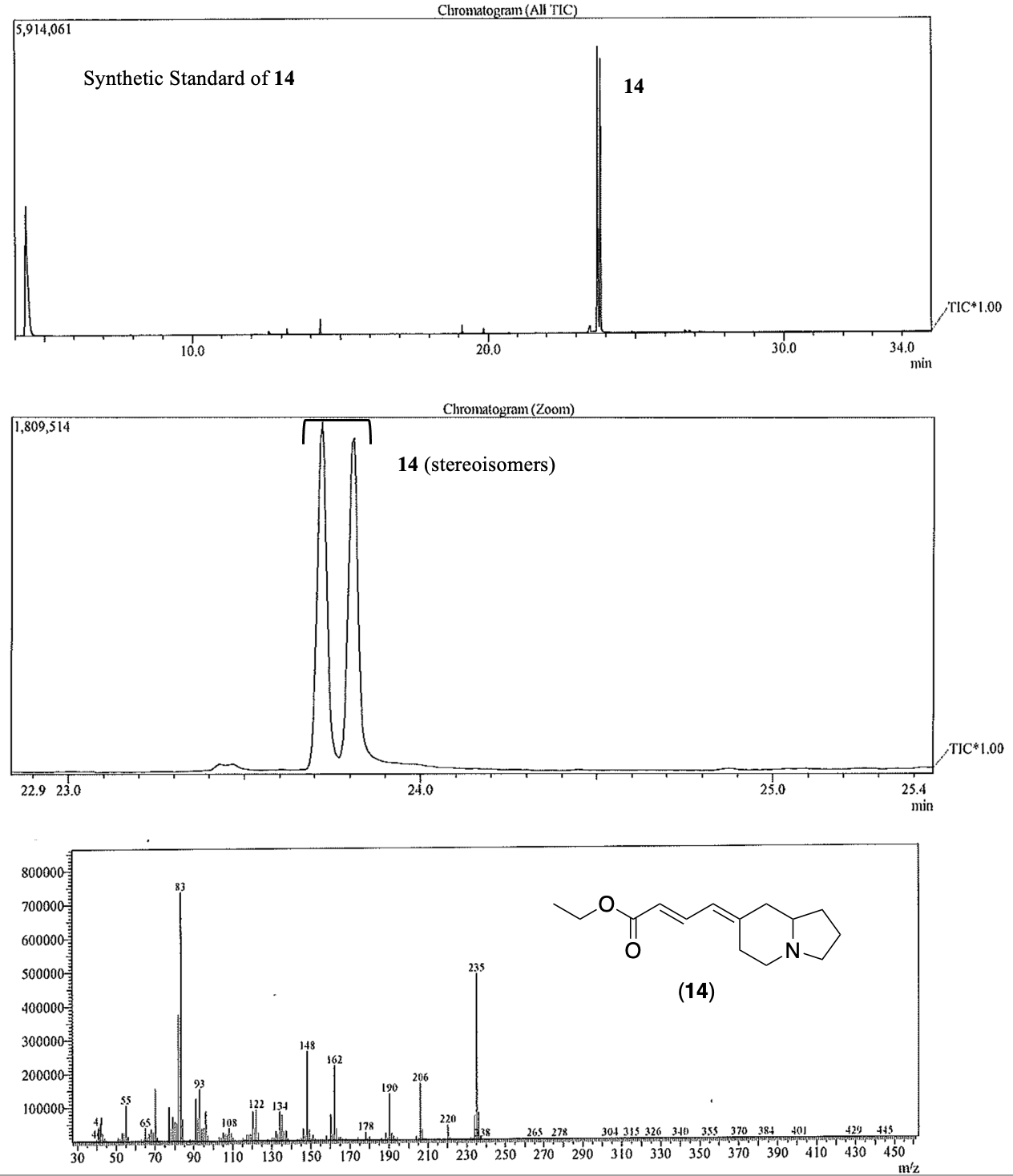
**

**Figure S20.** GCMS chromatogram and spectrum of the synthesized **14**

**
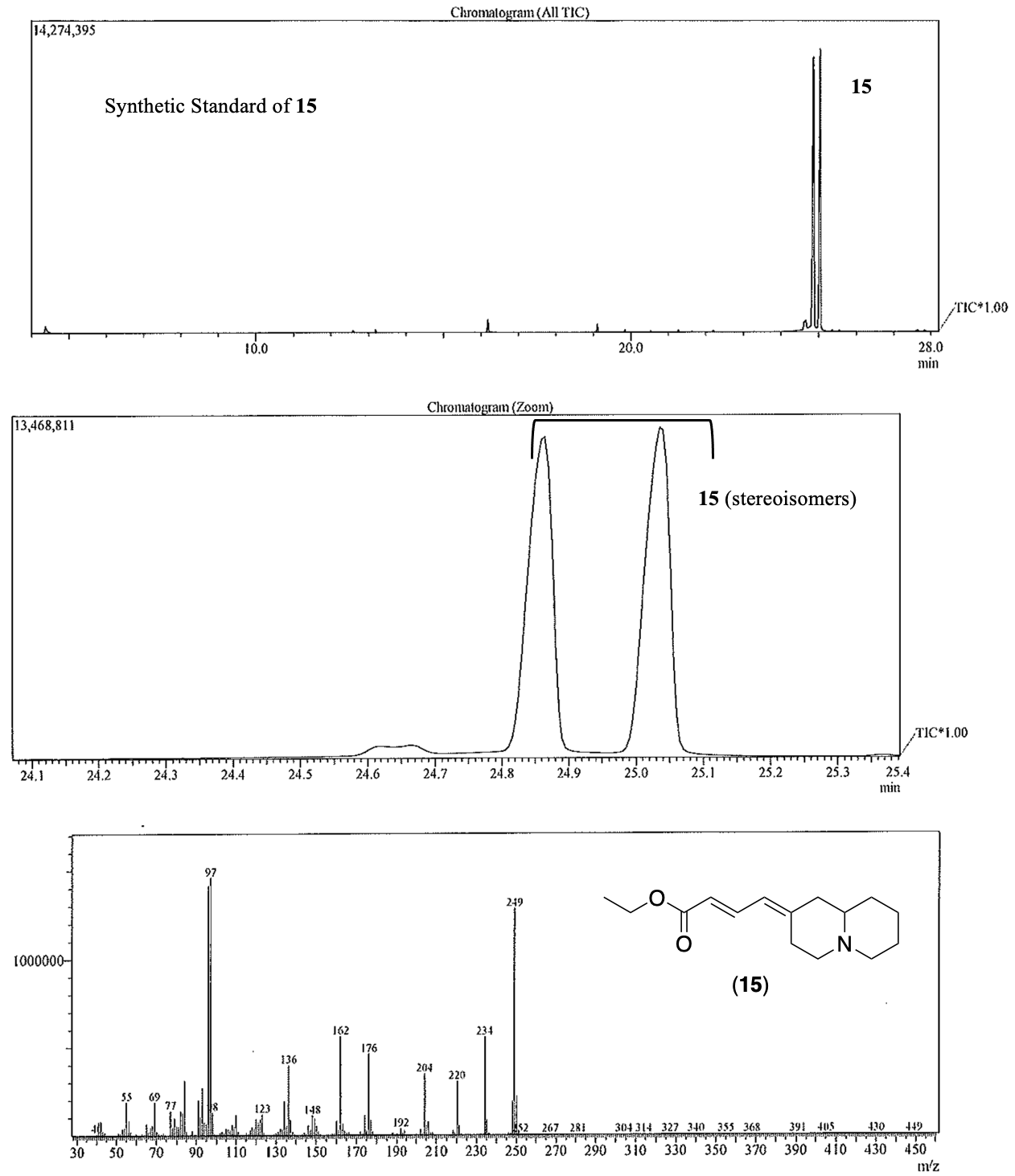
**

**Figure S21.** GCMS chromatogram and spectrum of the synthesized **15**

**
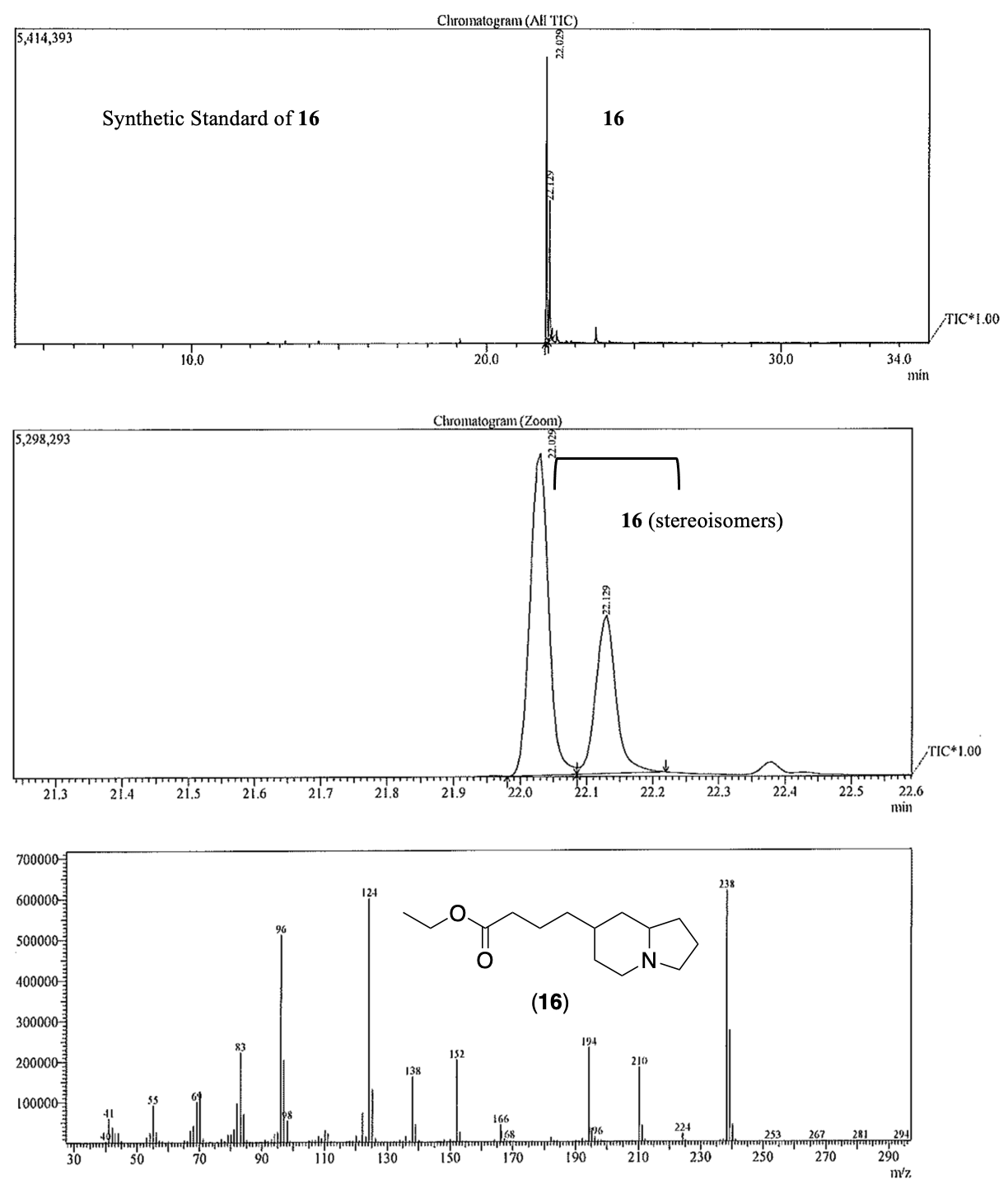
**

**Figure S22.** GCMS chromatogram and spectrum of the synthesized **16**

**
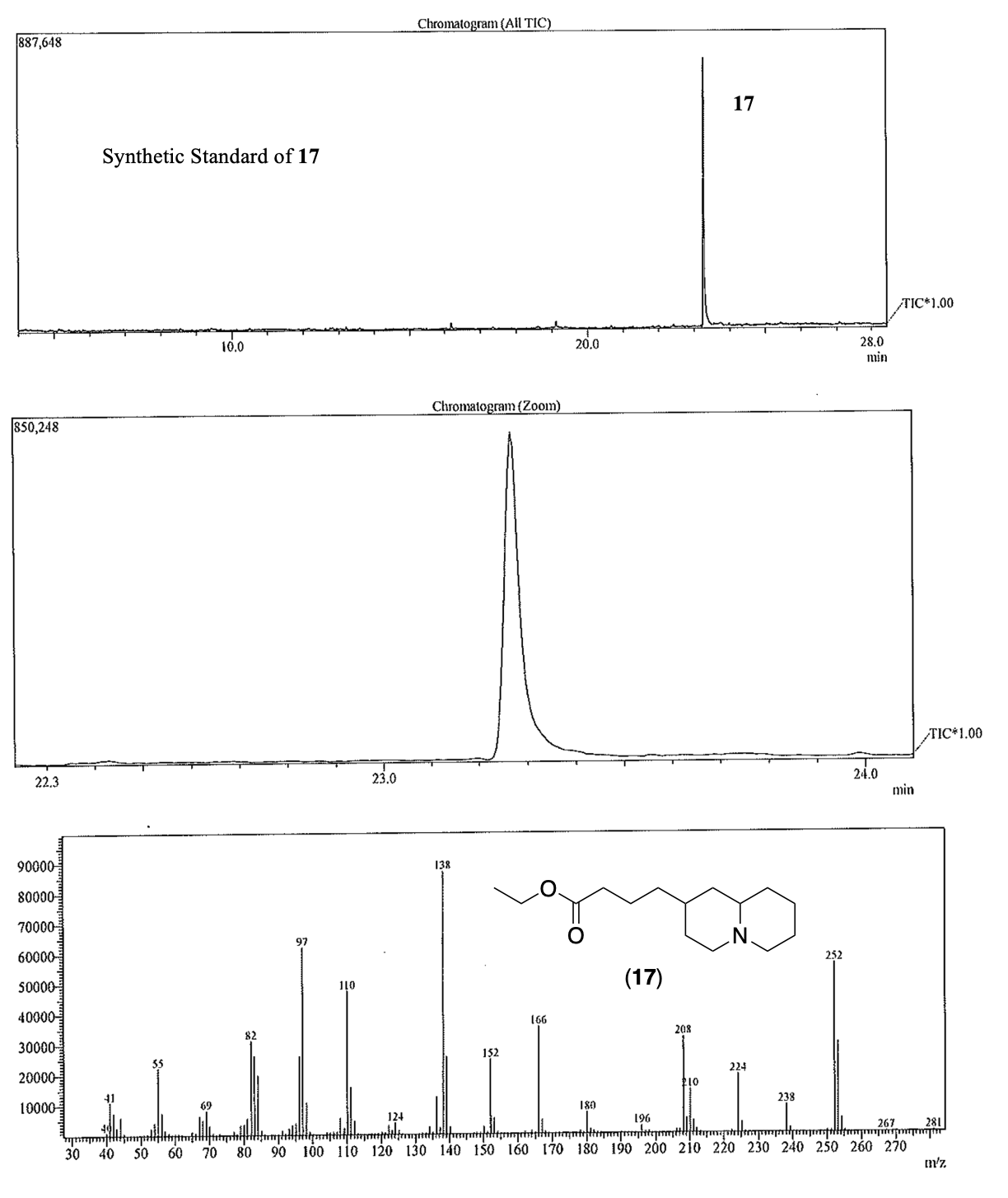
Figure S23.** GCMS chromatogram and spectrum of the synthesized **17**

**
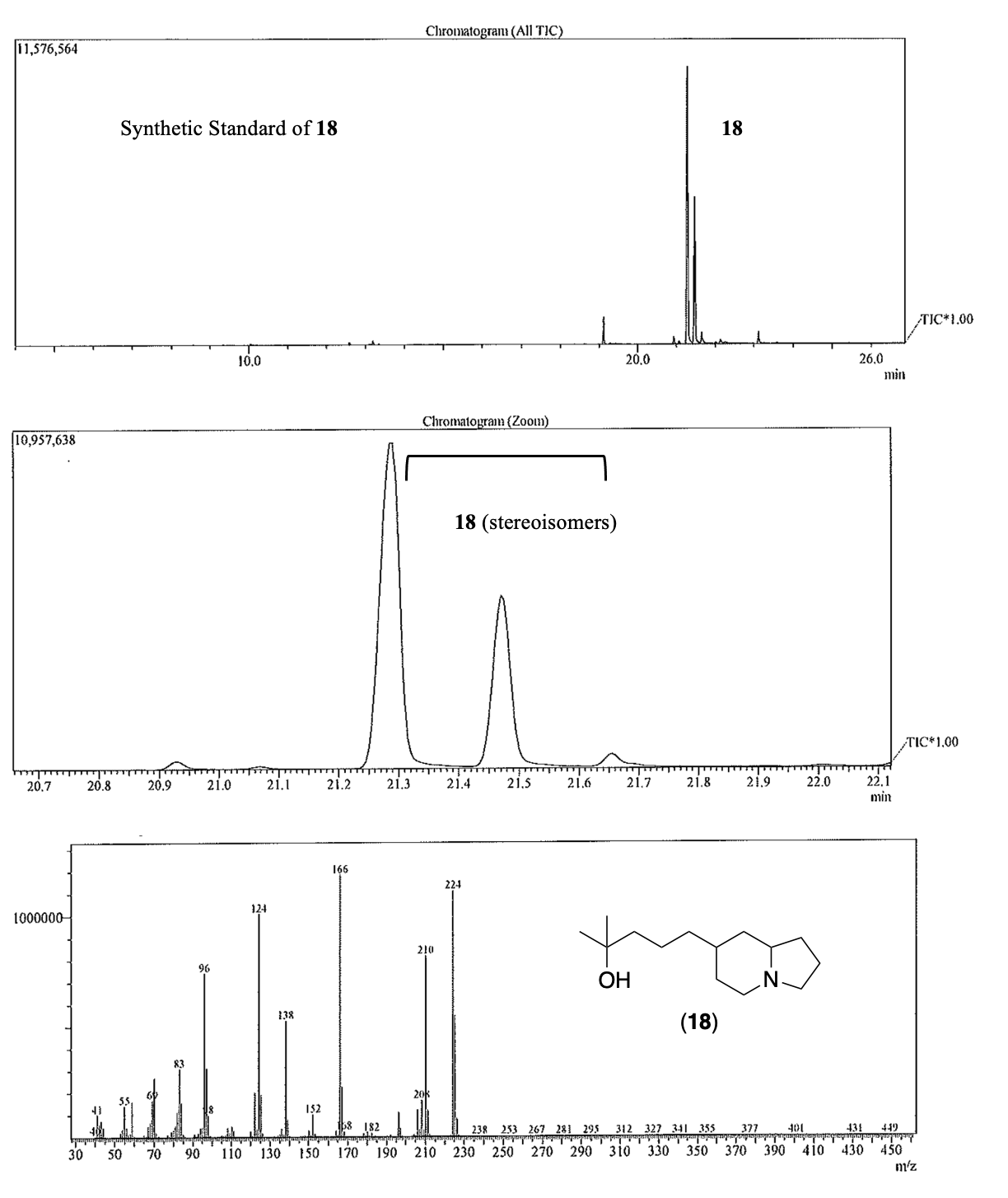
**

**Figure S24.** GCMS chromatogram and spectrum of the synthesized **18**

**
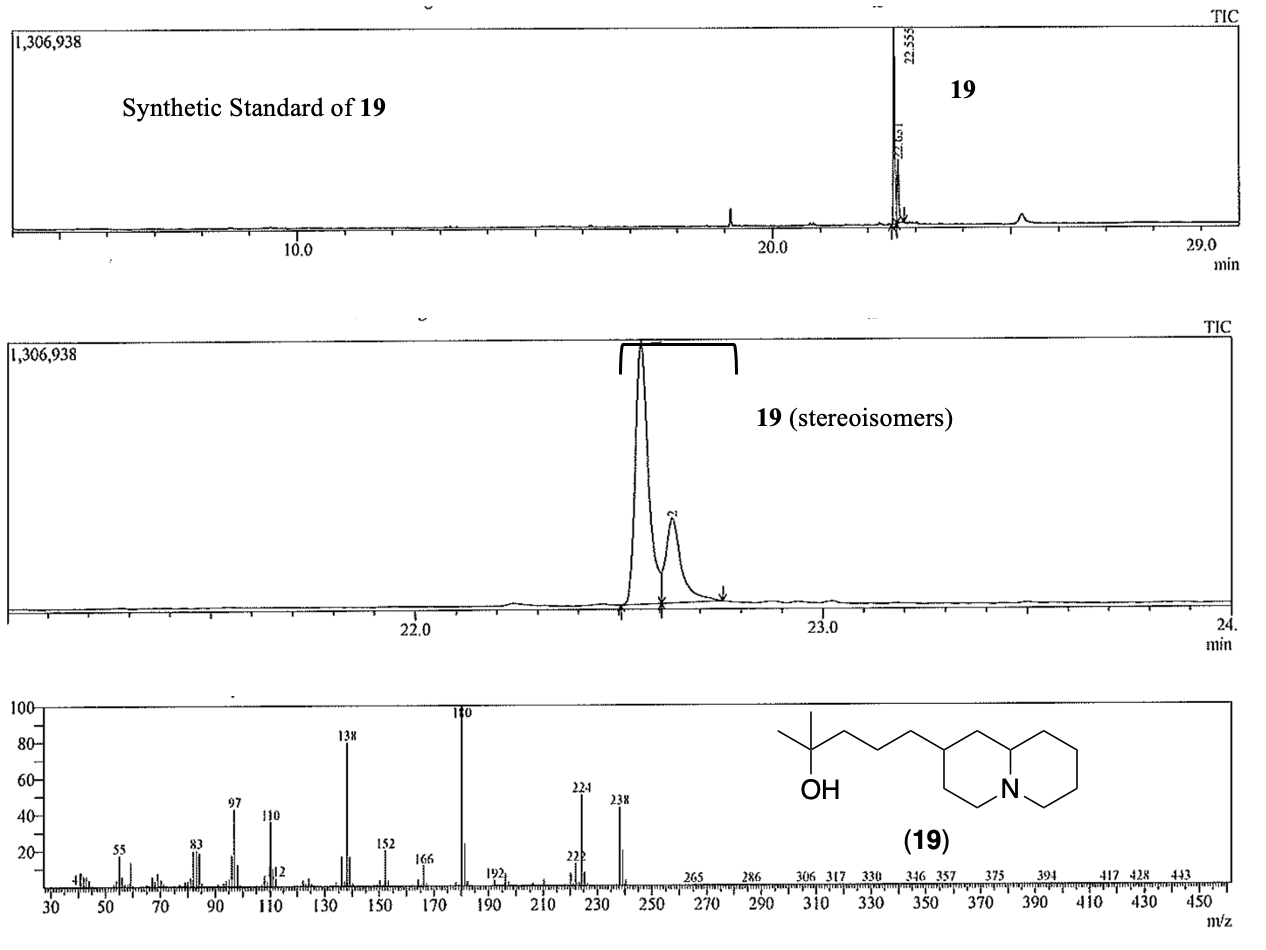
**

**Figure S25.** GCMS chromatogram and spectrum of the synthesized **19**


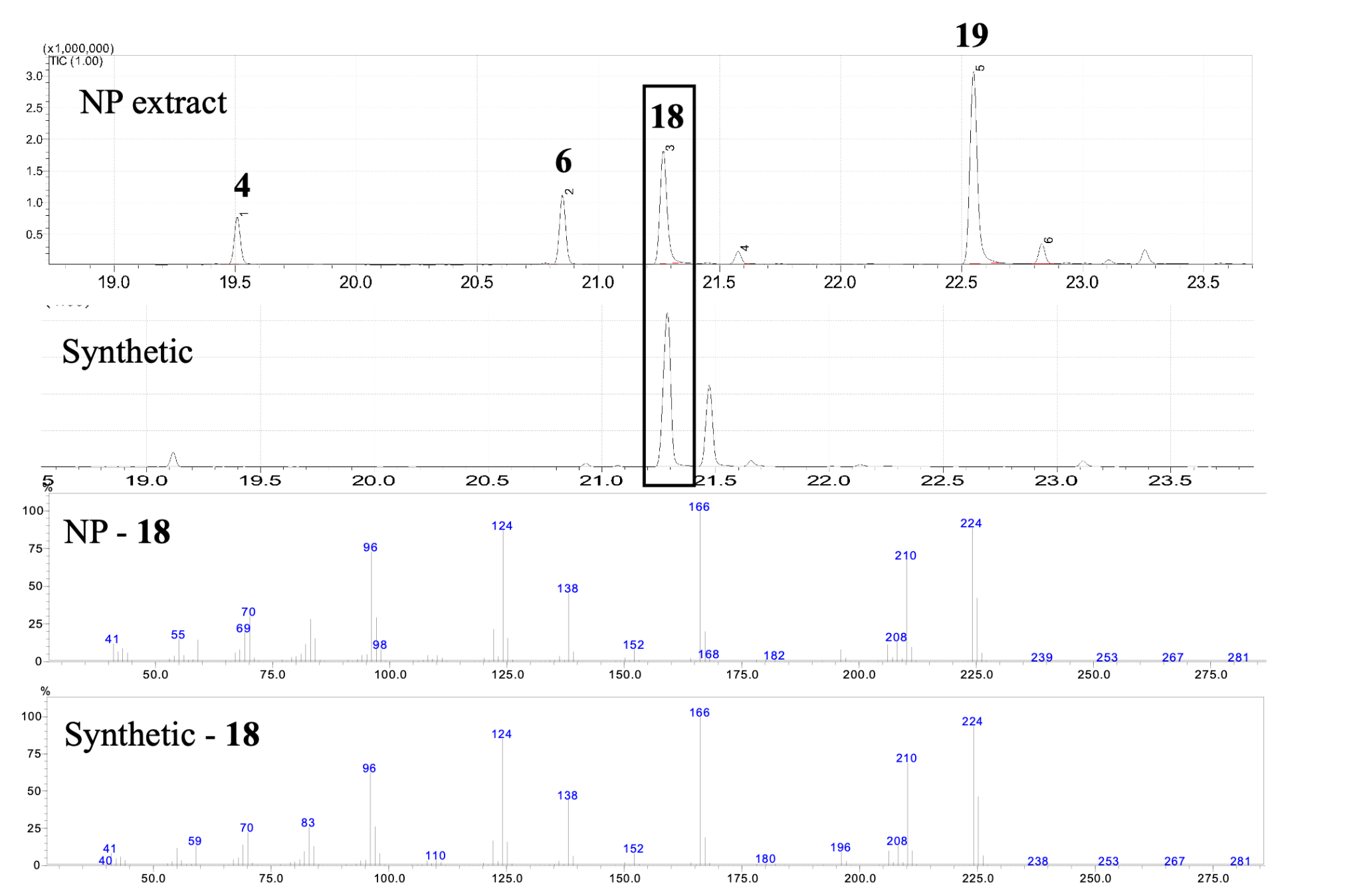


**Figure S26.** Comparison of synthetic **18** with hydratation of *B. petasata* extract. The synthesis is a mixture of isomers with the *syn-*isomer the major product (first eluting peak). The fragmentation patterns for the synthetic and natural material match.


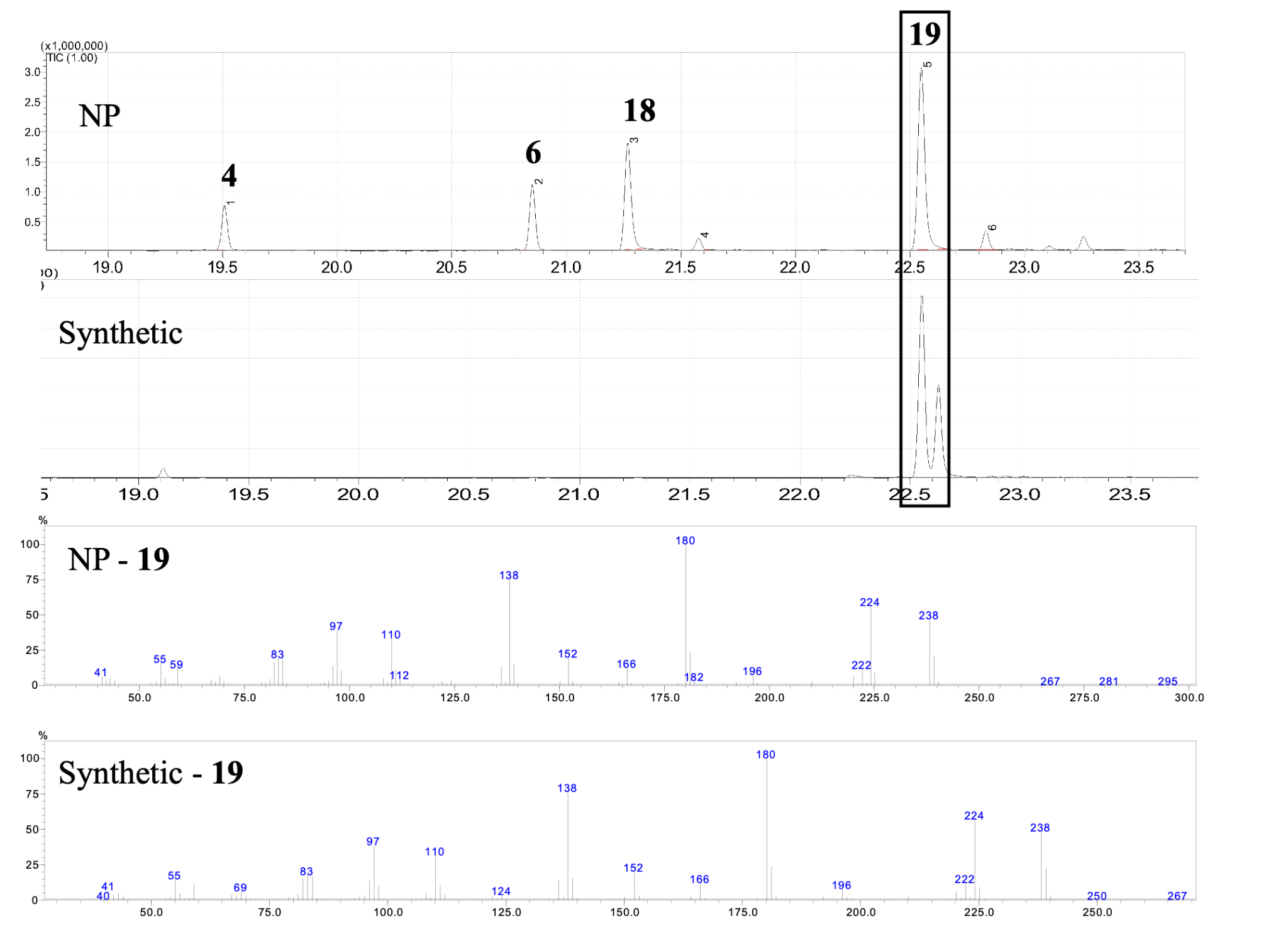


**Figure S27**. Comparison of synthetic **19** with hydratation of *B. petasata* extract. The synthesis is a mixture of isomers with the *syn-*isomer the major product (first eluting peak). The fragmentation patterns for the synthetic and natural material match.

| **Table S2.** NMR Spectroscopy Data [600 MHz, (CD_3_)_2_SO, 50^o^C] for the synthetic *syn-*isomer of **18** | | | | | |
| --- | --- | --- | --- | --- | --- |
| **position** | **δ_C_, type^[a]^** | **δ_H_ (J in Hz)** | **H2BC** | **HMBC** | **COSY** |
| 1 | 31.4, CH_2_ | 1.62, m | 2 |  | 2 |
|  |  | 1.14, m |  |  |  |
| 2 | 51.3, CH_2_ | 3.00, m |  |  | 1 |
|  |  | 1.97, m |  |  |  |
| 3 | 52.7, CH_2_ | 2.95, m | 4 | 2 | 4 |
|  |  | 2.04, m |  |  |  |
| 4 | 20.4, CH_2_ | 1.66, m | 3, 5 |  | 3 |
|  |  | 1.62, m |  |  |  |
| 5 | 29.6, CH_2_ | 1.78, m | 4, 6 |  |  |
|  |  | 1.27, m |  |  |  |
| 6 | 63.4, CH | 1.83, m | 5, 7 |  |  |
| 7 | 36.5, CH_2_ | 1.78, m | 6, 8 | 6 | 8 |
|  |  | 1.80, m |  |  |  |
| 8 | 35.4, CH | 1.28, m | 1 |  | 7 |
| 9 | 32.1, CH_2_ | 1.28, m |  |  |  |
| 10 | 21.4, CH_2_ | 1.29, m | 12 |  |  |
| 11 | 43.7, CH | 1.31, m | 10 | 10, 13, 14 |  |
| 12 | 68.2, C |  |  |  |  |
| 13 | 29.0, CH_3_ | 1.06, m |  | 11, 12, 14 |  |
| 14 | 29.0, CH_3_ | 1.06, m |  | 11, 12, 13 |  |

^[a]^δ_C_ obtained indirectly from gHSQC and gHMBC experiments.

| **Table S3.** NMR Spectroscopy Data [600 MHz, (CD_3_)_2_SO, 50^o^C] for the synthetic *anti-*isomer of **18** | | | | | |
| --- | --- | --- | --- | --- | --- |
| **Position** | **δ_C_, type^[a]^** | **δ_H_ (J in Hz)** | **H2BC** | **HMBC** | **COSY** |
| 1 | 28.6, CH_2_ | 1.71, m |  |  |  |
|  |  | 1.42, m |  |  |  |
| 2 | 46.8, CH_2_ | 2.79, m | 1 |  |  |
|  |  | 2.36, m |  |  |  |
| 3 | 52.8, CH_2_ | 2.95, m | 4 | 3 | 4 |
|  |  | 2.31, m |  |  |  |
| 4 | 19.9, CH_2_ | 1.71, m | 3, 5 | 5 | 3 |
|  |  | 1.66, m |  |  |  |
| 5 | 28.6, CH_2_ | 1.77, m | 4 |  |  |
|  |  | 1.36, m |  |  |  |
| 6 | 58.0, CH | 2.36, m | 5 |  |  |
| 7 | 33.2, CH_2_ | 1.62, m |  |  | 8 |
|  |  | 1.46, m |  |  |  |
| 8 | 30.1, CH | 1.71, m | 1 |  | 7 |
| 9 | 36.7, CH_2_ | 1.19, m | 10 |  |  |
| 10 | 20.7, CH_2_ | 1.31, m | 9, 11 |  |  |
| 11 | 43.6, CH | 1.32, m | 10 | 10, 13, 14 |  |
| 12 | 68.6, C |  |  |  |  |
| 13 | 28.6, CH_3_ | 1.06, m |  | 11, 12, 14 |  |
| 14 | 28.6, CH_3_ | 1.06, m |  | 11, 12, 13 |  |

^[a]^δ_C_ obtained indirectly from gHSQC and gHMBC experiments.


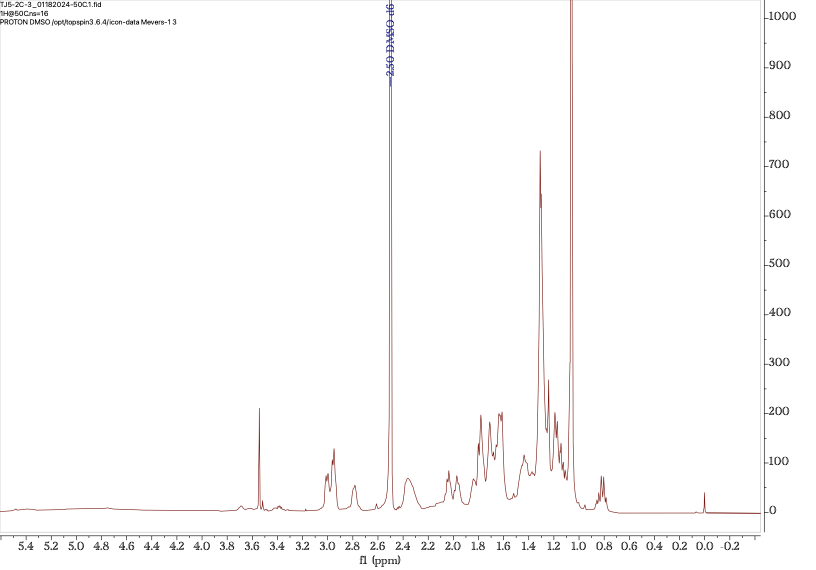


**Figure S28.** ^1^H NMR spectrum for compound **18** (600 MHz, *d_6_*-DMSO). 16 scans, 50^o^C


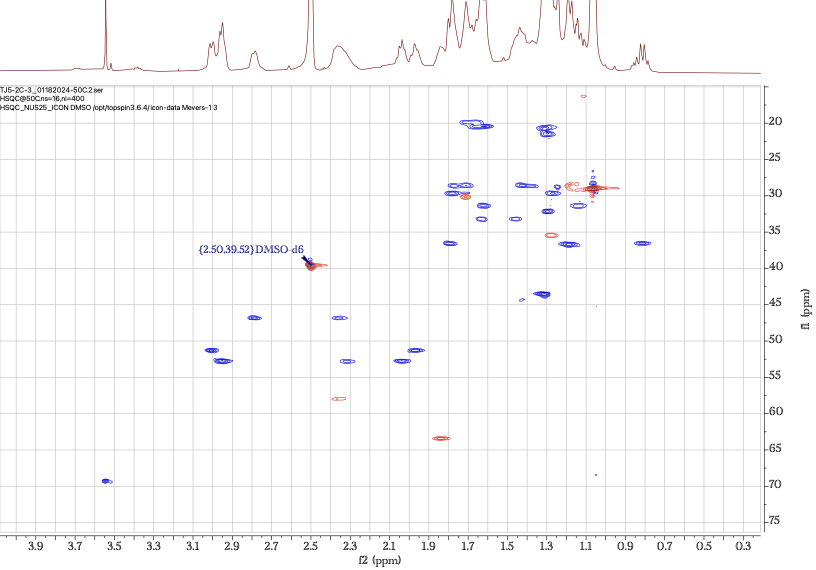


**Figure S29.** gHSQC for compound **18** (600 MHz, *d_6_*-DMSO). 16 scans, NUS25, 400 increments, and 50^o^C


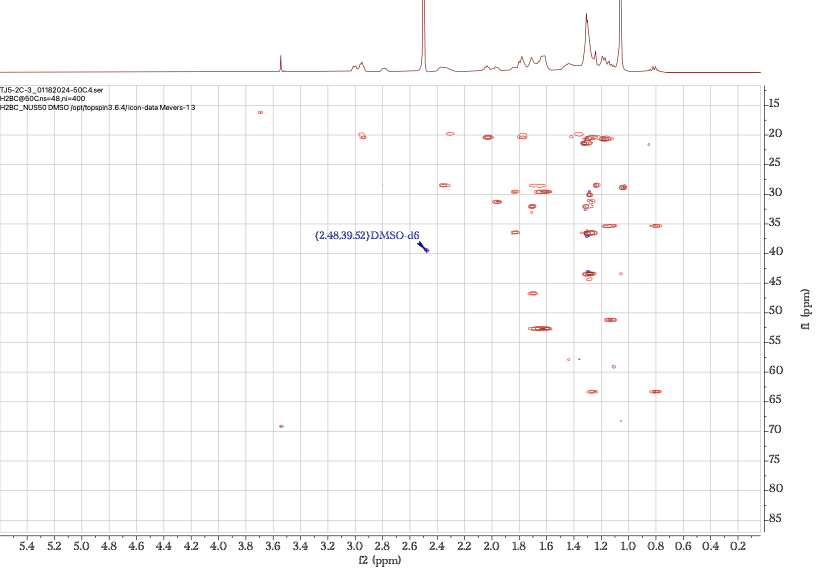


**Figure S30.** H2BC for compound **18** (600 MHz, *d_6_*-DMSO). 48 scans, NUS50, 400 increments, and 50^o^C


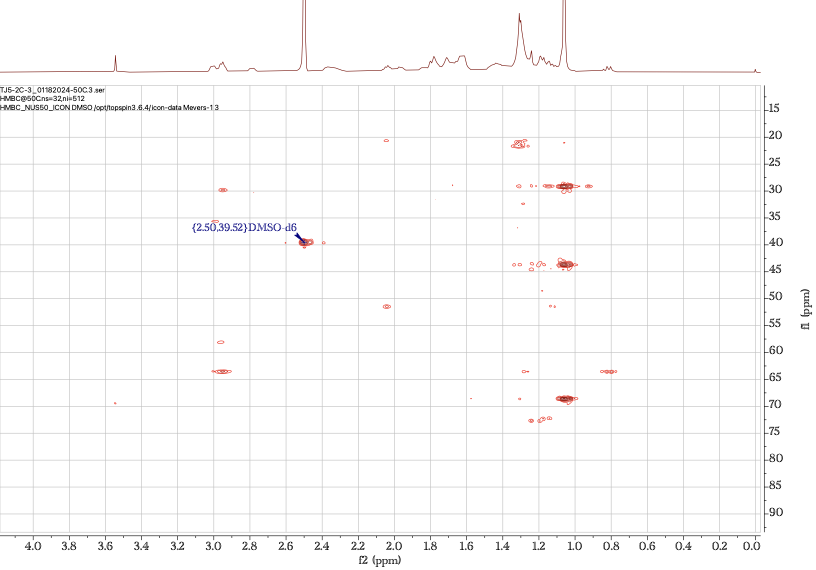


**Figure S31.** HMBC for compound **18** (600 MHz, *d_6_*-DMSO). 32 scans, NUS50, 512 increments, and 50^o^C


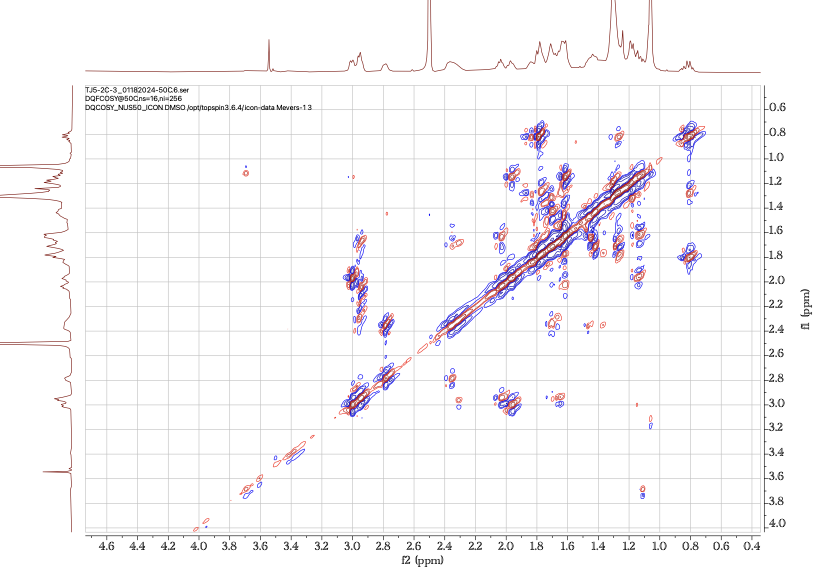


**Figure S32.** dqfCOSY for compound **18** (600 MHz, *d_6_*-DMSO). 16 scans, NUS50, and 400 increments, and 50^o^C

**
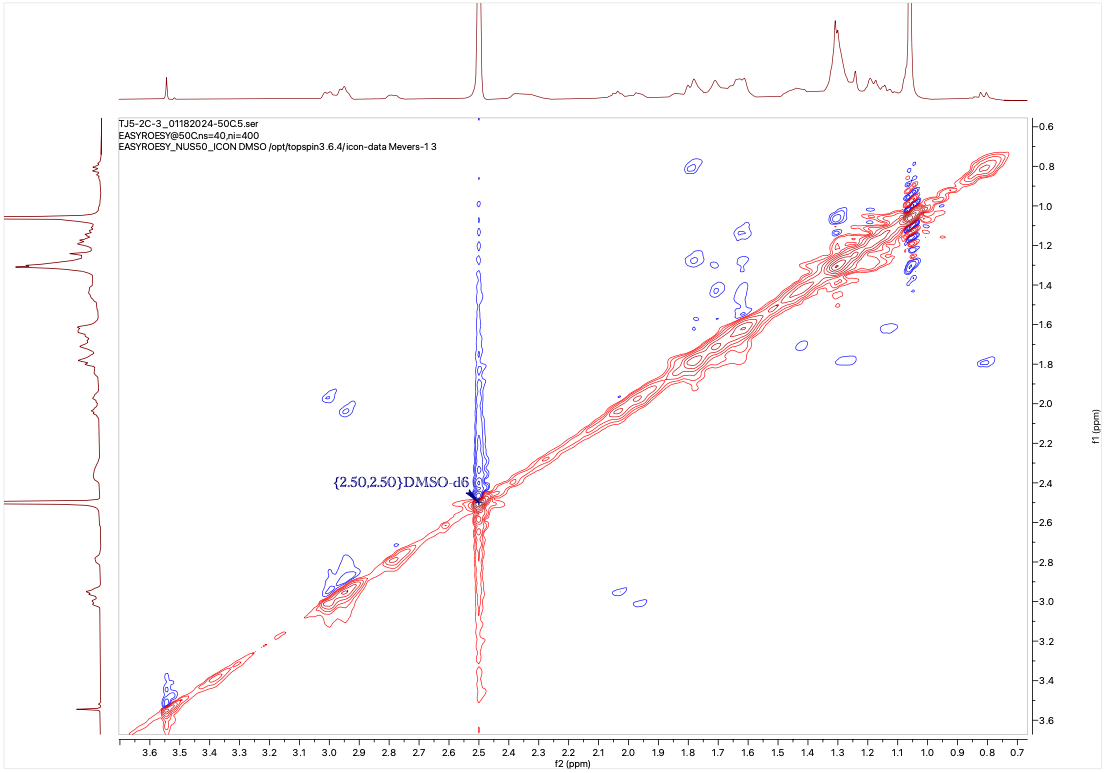
**

**Figure S33.** easyROESY for compound **18** (600 MHz, *d_6_*-DMSO). 40 scans, NUS50, 400 increments, 400 μsec mixing time, and 50^o^C.

| **Table S4.** NMR Spectroscopy Data [600 MHz, (CD_3_)_2_SO, 50^o^C] for the synthetic *syn-*isomer of compound **19** | | | | | | |
| --- | --- | --- | --- | --- | --- | --- |
| **Position** | **δ_C_, type^[a]^** | **δ_H_ (J in Hz)** | **H2BC** | **HMBC** | **COSY** | **ROESY** |
| 1 | 31.4, CH_2_ | 1.69, m | 2 |  | 2 |  |
|  |  | 1.27, m |  |  |  |  |
| 2 | 51.3, CH_2_ | 3.01, m | 1 |  | 1, 6, 14, 15 |  |
|  |  | 2.40, m |  |  |  |  |
| 3 | 52.7, CH_2_ | 3.01, m |  |  | 5 |  |
|  |  | 2.36, m |  |  |  |  |
| 4 | 20.4, CH_2_ | 1.69, m |  |  |  |  |
|  |  | 1.30, m |  |  |  |  |
| 5 | 23.5, CH_2_ | 1.64, m |  |  | 3 |  |
| 6 | 30.7, CH_2_ | 1.64, m | 5, 7 | 5 | 7 |  |
|  |  | 1.35, m |  |  |  |  |
| 7 | 61.6, CH | 2.35, m | 6, 8 |  | 6, 14, 15 | 9 |
| 8 | 37.4, CH_2_ | 1.66, m | 7, 9 |  | 14, 15 |  |
|  |  | 1.04, m |  |  |  |  |
| 9 | 34.1, CH | 1.41, m | 1, 8 | 11, 12 | 14, 15 | 7 |
| 10 | 36.1, CH_2_ | 1.15, m | 9, 11 | 11. 12 |  |  |
| 11 | 20.4, CH_2_ | 1.30, m | 10, 12 | 12 |  |  |
| 12 | 43.4, CH | 1.31, m | 11 | 11, 13, 14, 15 |  |  |
| 13 | 68.6, C |  |  |  |  |  |
| 14 | 28.9, CH_3_ | 1.06, m |  | 7, 12, 13, 15 | 7, 8, 9 |  |
| 15 | 28.9, CH_3_ | 1.06, m |  | 7, 12, 13, 14 | 7, 8, 9 |  |
| ^[a]^δ_C_ obtained indirectly from gHSQC and gHMBC experiments. | | | | | | |

| **Table S5.** NMR Spectroscopy Data [600 MHz, (CD_3_)_2_SO, 50^o^C] for the synthetic *anti-*isomer of compound **19** | | | | | |
| --- | --- | --- | --- | --- | --- |
| **Position** | **δ_C_, type^[a]^** | **δ_H_ (J in Hz)** | **H2BC** | **HMBC** | **COSY** |
| 1 | 26.9, CH_2_ | 1.88, m | 2 |  | 2 |
|  |  | 1.52, m |  |  |  |
| 2 | 48.3, CH_2_ | 2.91, m | 1 |  | 1 |
|  |  | 2.67, m |  |  |  |
| 3 | 53.5, CH_2_ | 3.03, m |  |  |  |
|  |  | 2.61, m |  |  |  |
| 4 | 22.4, CH_2_ | 1.64, m |  |  |  |
|  |  | 1.39, m |  |  |  |
| 5 | 23.4, CH_2_ | 1.60, m |  |  |  |
| 6 | 30.8, CH_2_ | 1.64, m | 5, 7 | 5 |  |
|  |  | 1.32, m |  |  |  |
| 7 | 56.5, CH | 2.73, m | 8 |  |  |
| 8 | 34.4, CH_2_ | 1.62, m | 7, 9 |  | 14, 15 |
|  |  | 1.53, m |  |  |  |
| 9 | 29.3, CH | 1.73, m |  |  |  |
| 10 | 36.2, CH_2_ | 1.15, m |  | 12 |  |
| 11 | 21.5, CH_2_ | 1.29, m | 10, 12 | 12 |  |
| 12 | 43.3, CH | 1.34, m | 11 | 11, 13, 14, 15 |  |
| 13 | 68.5, C |  |  |  |  |
| 14 | 28.5, CH_3_ | 1.06, m |  | 12, 13, 15 | 8 |
| 15 | 28.5, CH_3_ | 1.06, m |  | 12, 13, 14 | 8 |
| ^[a]^δ_C_ obtained indirectly from gHSQC and gHMBC experiments. | | | | | |


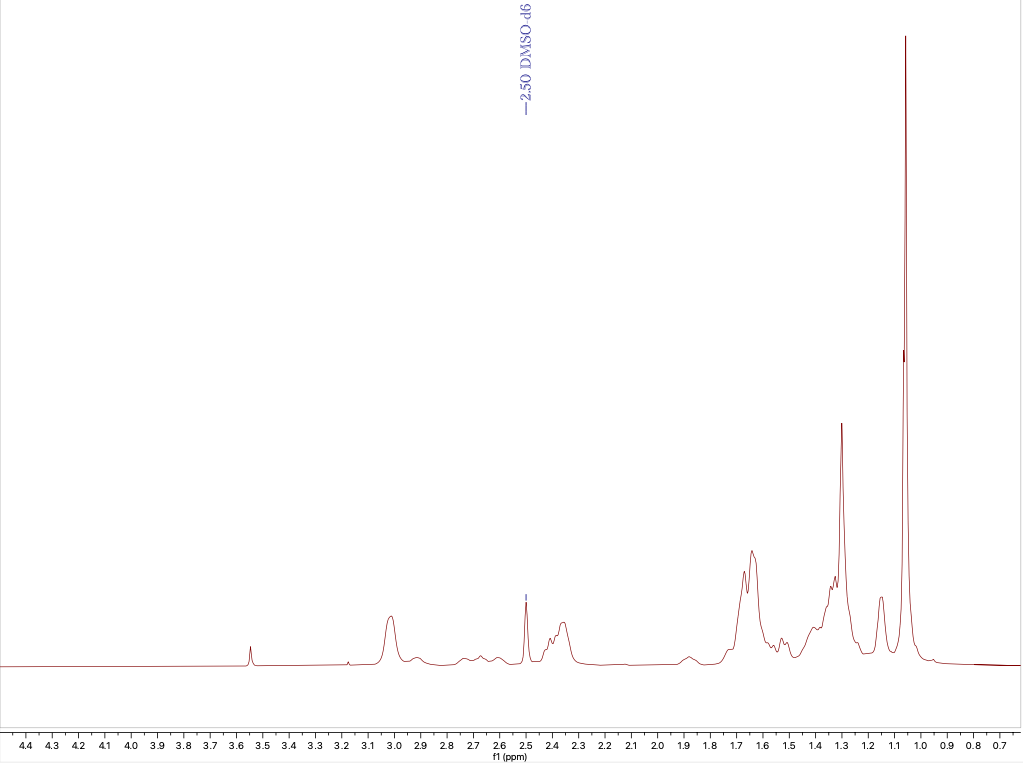


**Figure S34.** ^1^H NMR spectrum for compound **19** (600 MHz, *d_6_*-DMSO). 16 scans, 50^o^C


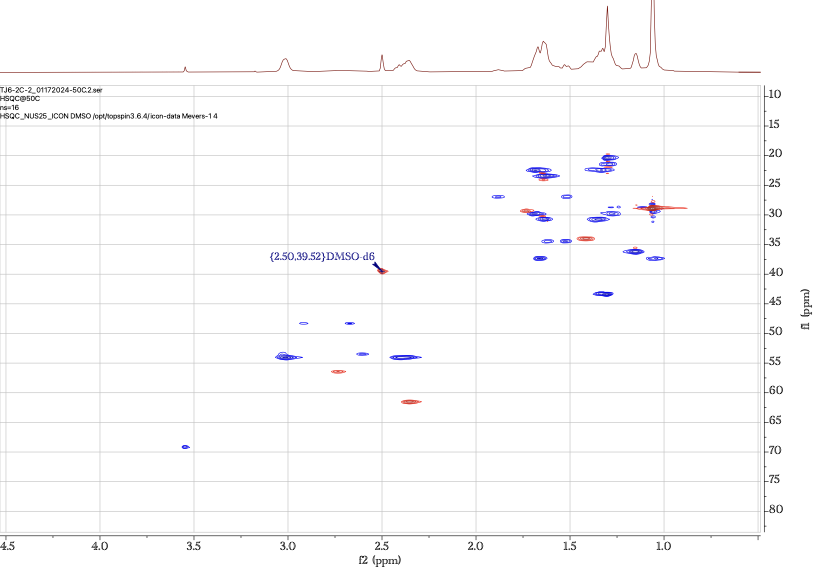


**Figure S35.** gHSQC for compound **19** (600 MHz, *d_6_*-DMSO). 16 scans, NUS25, 400 increments, and 50^o^C


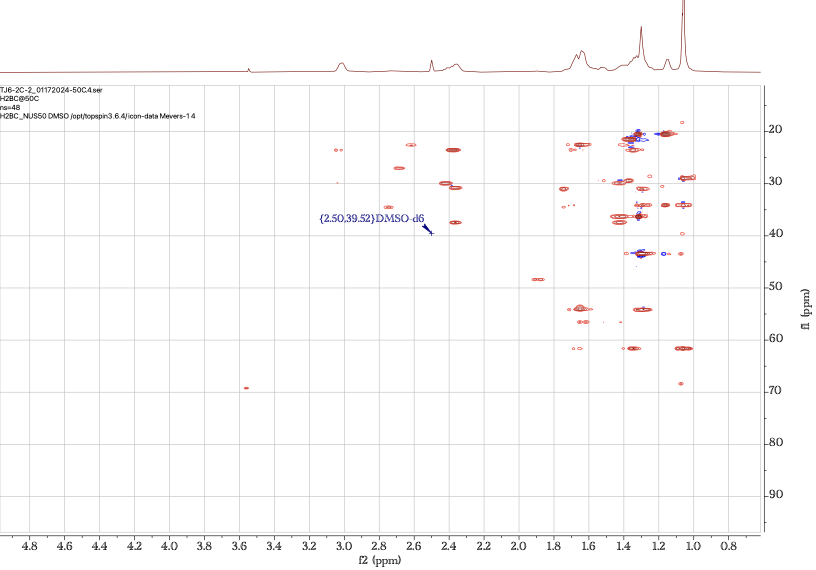


**Figure S36.** H2BC for compound **19** (600 MHz, *d_6_*-DMSO). 48 scans, NUS50, 400 increments, and 50^o^C


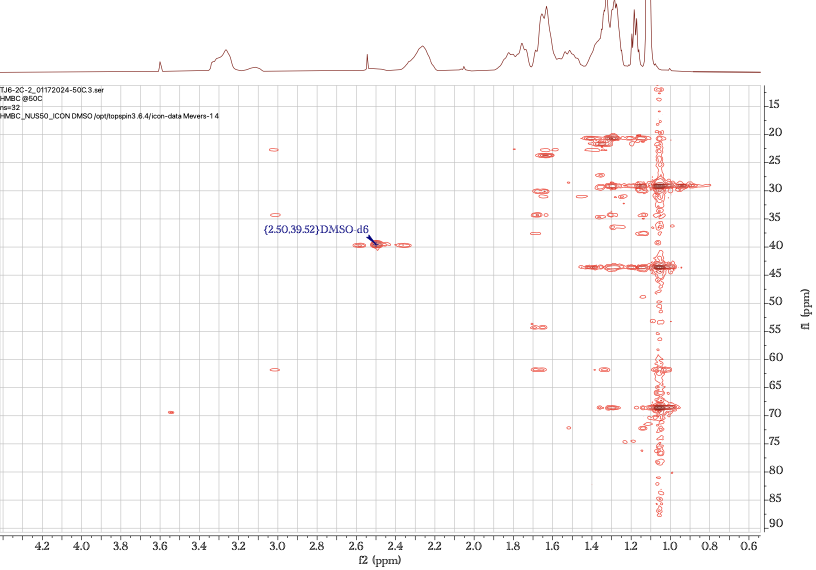


**Figure S37.** HMBC for compound **19** (600 MHz, *d_6_*-DMSO). 32 scans, NUS50, 512 increments, and 50^o^C


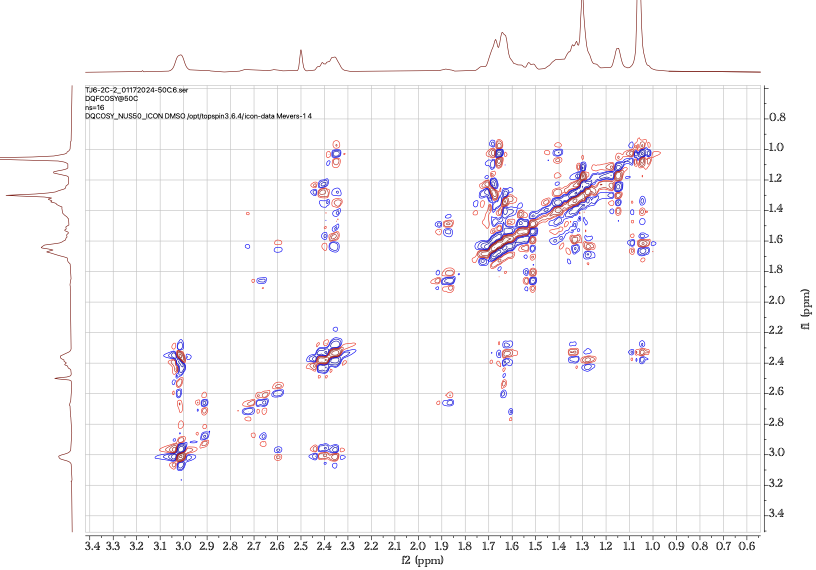


**Figure S38.** dqfCOSY for compound **19** (600 MHz, *d_6_*-DMSO). 16 scans, NUS50, and 400 increments, and 50^o^C


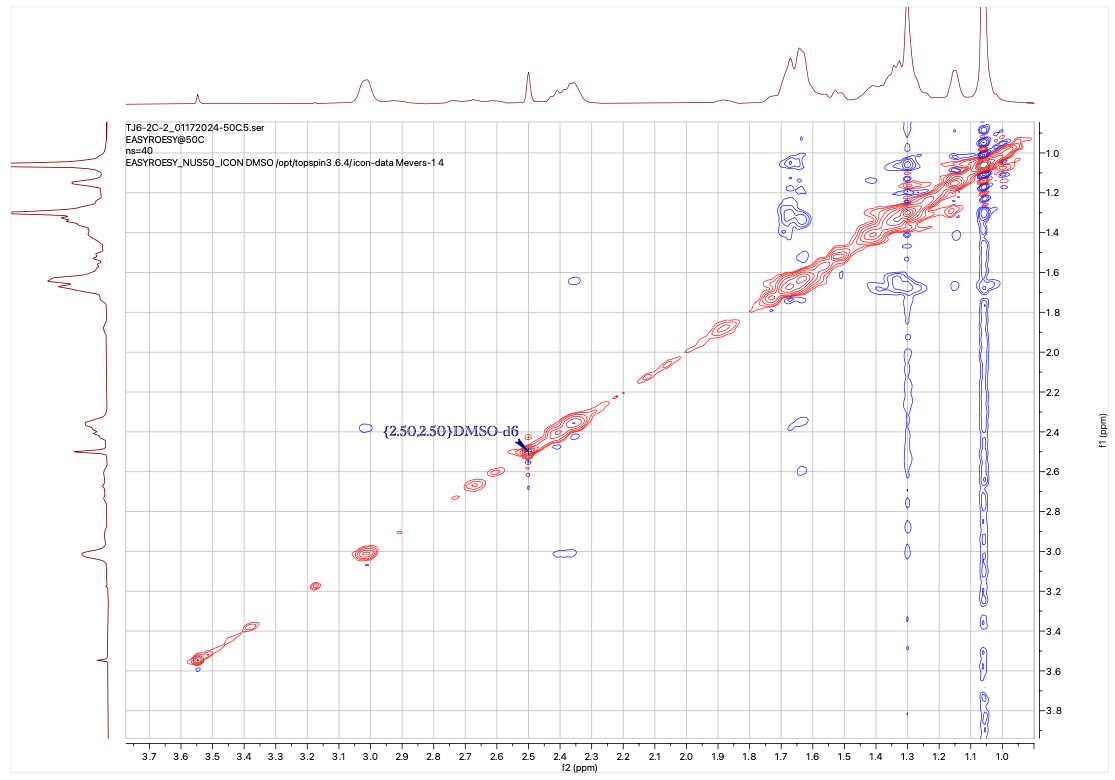


**Figure S39.** easyROESY for compound **19** (600 MHz, *d_6_*-DMSO). 40 scans, NUS50, 400 increments, 400 μsec mixing time, and 50^o^C.

| **Table S6.** NMR Spectroscopy Data [600 MHz, (CD_3_)_2_SO, 50^o^C] for natural hydrogosodesmine (**4**) | | | | | |
| --- | --- | --- | --- | --- | --- |
| **Position.** | **δ_C_, type^[a]^** | **δ_H_ (J in Hz)** | **H2BC** | **HMBC** | **ROESY** |
| 1 | 29.8, CH_2_ | 1.75, m |  |  |  |
|  |  | 1.23, m |  |  |  |
| 2 | 51.5, CH_2_ | 2.97, m |  |  |  |
|  |  | 1.88, m |  |  |  |
| 3 | 53.0, CH_2_ | 2.90, m | 4 |  |  |
|  |  | 1.96, m |  |  |  |
| 4 | 20.4, CH_2_ | 1.64, m |  |  |  |
|  |  | 1.59, m |  |  |  |
| 5 | 28.5, CH_2_ | 1.25, m |  |  |  |
| 6 | 63.3, CH | 1.71, m |  |  |  |
| 7 | 36.8, CH_2_ | 1.77, m |  |  |  |
|  |  | 0.78, m |  |  |  |
| 8 | 31.4, CH | 2.10, n |  |  |  |
| 9 | 36.1, CH_2_ | 1.23, n | 10 |  |  |
| 10 | 24.4, CH_2_ | 1.96, m |  | 9 |  |
| 11 | 124.2, CH | 5.09, m | 10 |  | 14 |
| 12 | 130.3, C |  |  |  |  |
| 13 | 17.1, CH_3_ | 1.57, s |  | 11, 12, 14 |  |
| 14 | 25.0, CH_3_ | 1.65, s |  | 11, 12, 13 | 11 |

^[a]^δ_C_ obtained indirectly from gHSQC and gHMBC experiments.


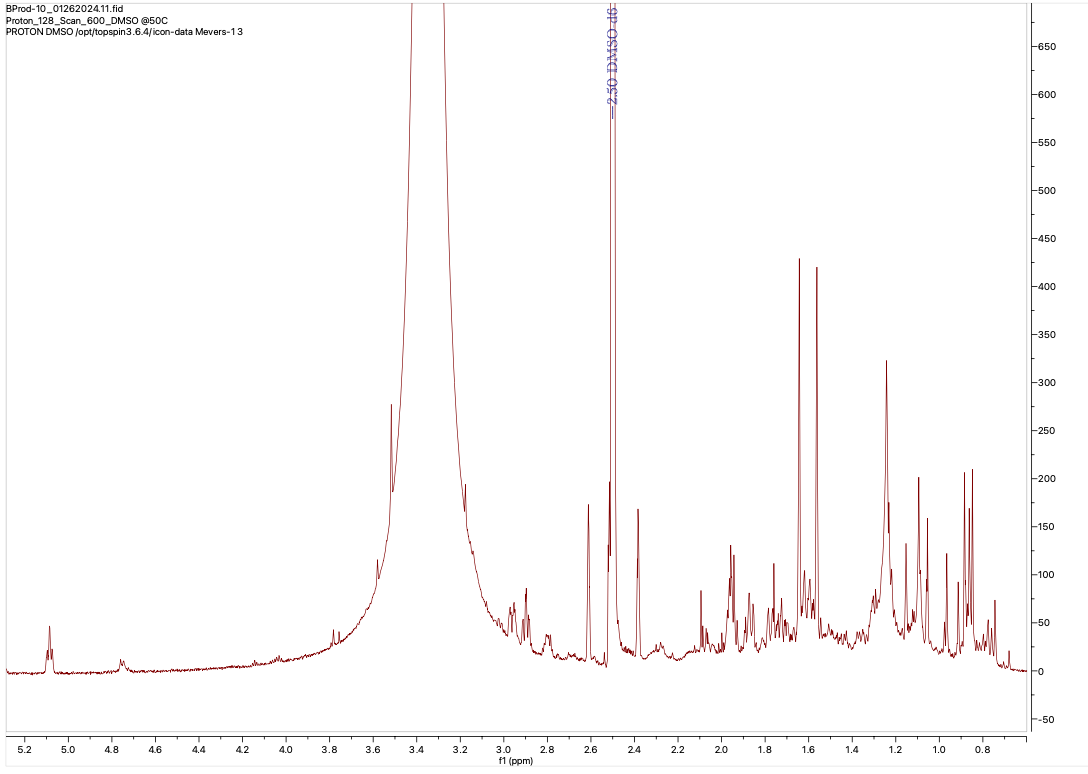


**Figure S40**. ^1^H NMR spectrum for hydrogosodesmine (**4**) (600 MHz, *d_6_*-DMSO). 128 scans, 50^o^C


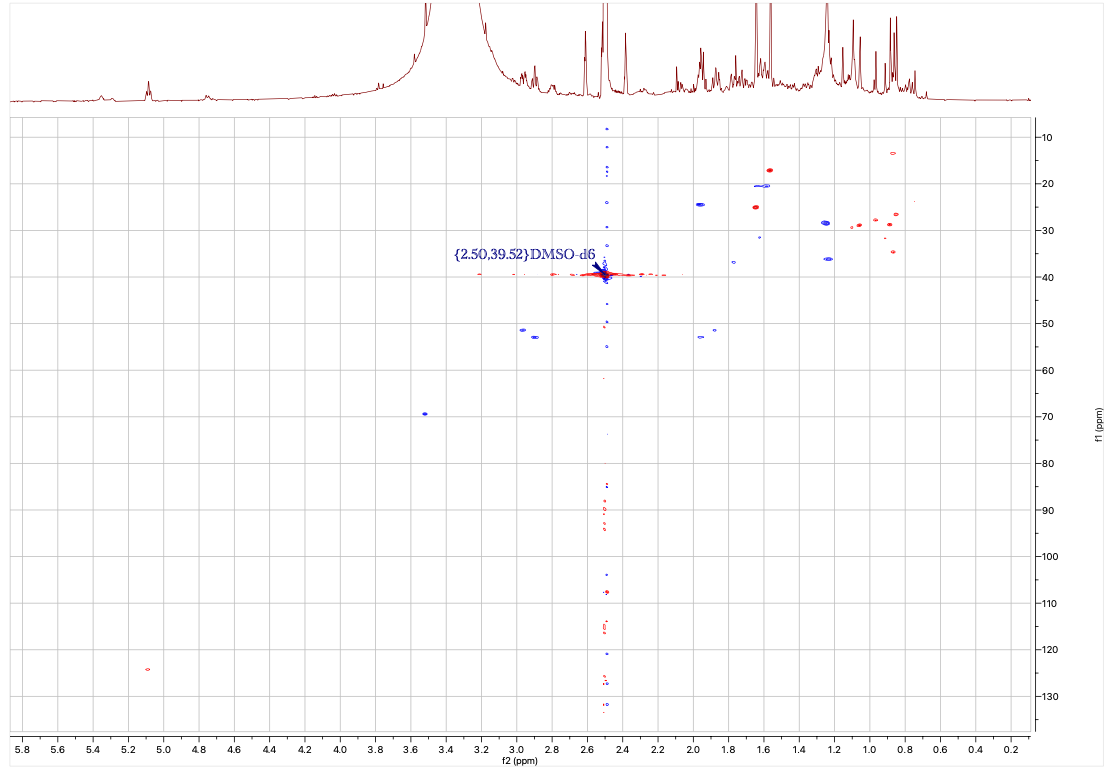


**Figure S41**. gHSQC for hydrogosodesmine (**4**) (600 MHz, *d_6_*-DMSO). 96 scans, NUS25, 400 increments


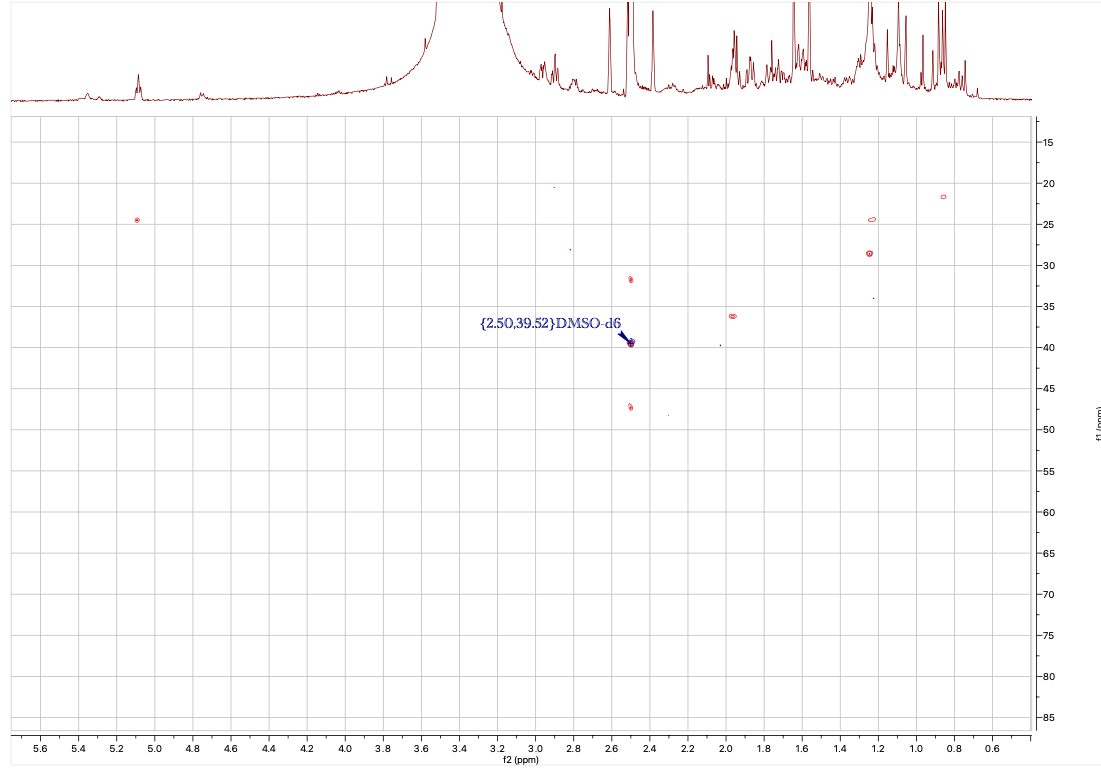


**Figure S42.** H2BC for hydrogosodesmine (**4**) (600 MHz, *d_6_*-DMSO). 160 scans, NUS50, 400 increments, and 50^o^C


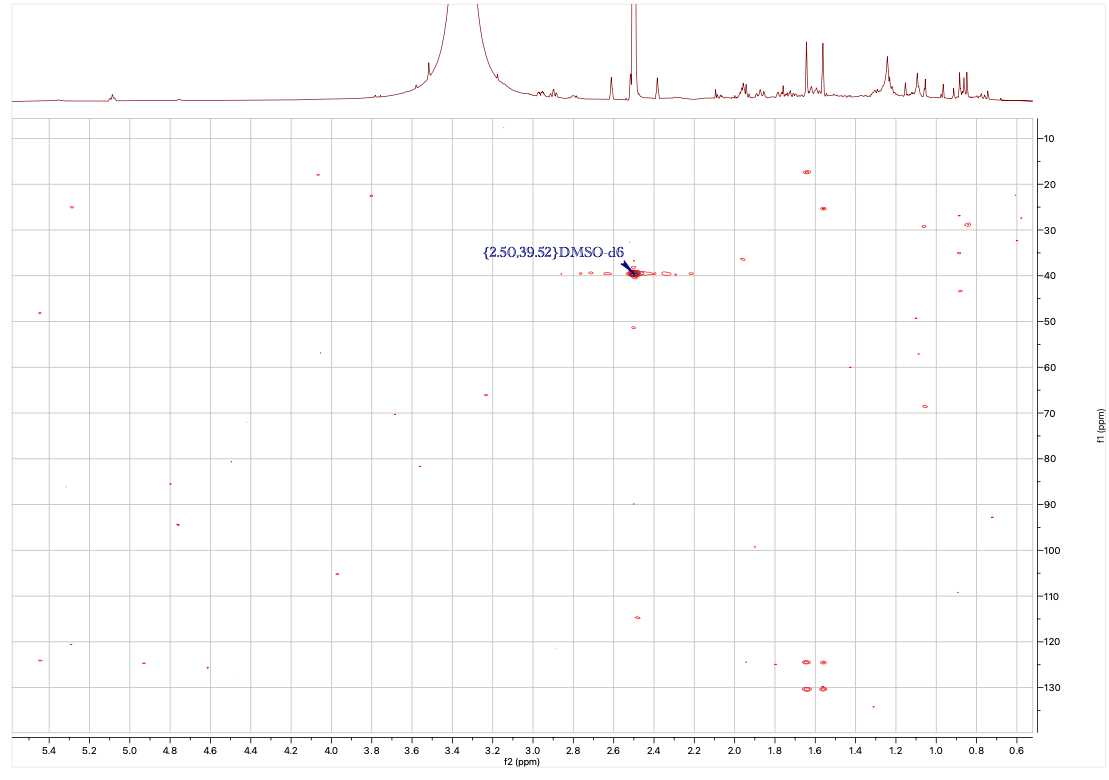


**Figure S43.** HMBC for hydrogosodesmine (**4**) (600 MHz, *d_6_*-DMSO). 64 scans, NUS50, and 512 increments


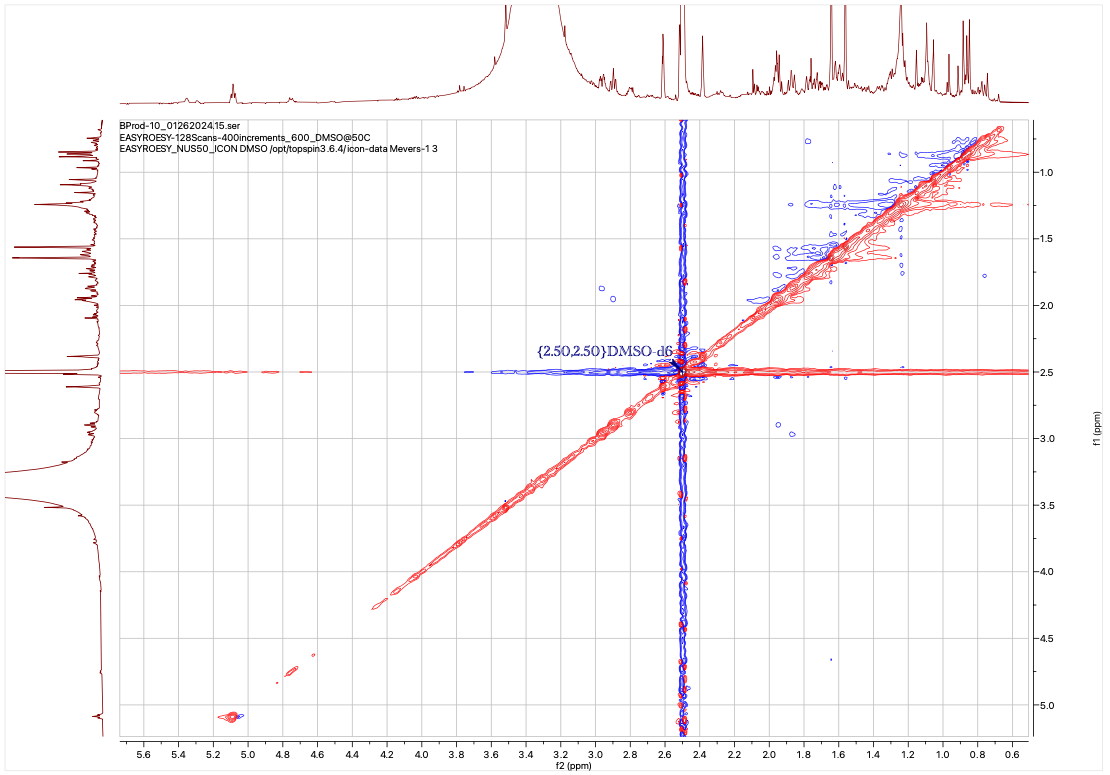


**Figure S44.** easyROESY for hydrogosodesmine (**4**) (600 MHz, *d_6_*-DMSO). 128 scans, NUS50, 400 increments, and 400 μsec mixing time.

| **Table S7.** NMR Spectroscopy Data [600 MHz, (CD_3_)_2_SO, 50^o^C] for natural homo-hydrogosodesmine (**6**) | | | | | | |
| --- | --- | --- | --- | --- | --- | --- |
| **Position** | **δ_C_, type^[a]^** | **δ_H_ (J in Hz)** | **H2BC** | **HMBC** | **COSY** | **ROESY** |
| 1 | 31.6, CH_2_ | 1.59, m | 2 |  |  |  |
|  |  | 1.08, m |  |  |  |  |
| 2 | 55.46, CH_2_ | 2.72, m | 1 |  | 3 | 1, 3 |
|  |  | 1.88, m |  |  |  |  |
| 3 | 55.44, CH_2_ | 2.70, m | 4 |  | 2 | 2 |
|  |  | 1.92, m |  |  |  |  |
| 4 | 25.2, CH_2_ | 1.53, m | 3 |  |  |  |
|  |  | 1.42, m |  |  |  |  |
| 5 | 23.9, CH_2_ | 1.61, m | 4 |  |  |  |
|  |  | 1.17, m |  |  |  |  |
| 6 | 32.6, CH_2_ | 1.48, m | 7 | 5 |  |  |
|  |  | 1.10, m |  |  |  |  |
| 7 | 61.6, CH | 1.67, m | 6, 8 |  |  |  |
| 8 | 39.4, CH_2_ | 1.53, m | 9, 7 |  |  |  |
|  |  | 0.80, m |  |  |  |  |
| 9 | 34.9, CH | 1.24, m | 1, 8 |  | 11 |  |
| 10 | 36.4, CH_2_ | 1.16, m | 11 | 1, 8, 9, 12, 11 | 11 | 11 |
| 11 | 24.5, CH_2_ | 1.92, m | 10, 12 | 10, 12 | 9, 11, 12 | 10, 12, 14, 15 |
| 12 | 124.4, CH | 5.06, m | 11 | 13, 14 | 11 | 11, 15 |
| 13 | 130.6, C |  |  |  |  |  |
| 14 | 17.5, CH_3_ | 1.54, s |  | 12, 13, 15 |  | 11 |
| 15 | 25.4, CH_3_ | 1.62, s |  | 12, 13, 14 |  | 11, 12 |

^[a]^δ_C_ obtained indirectly from gHSQC and gHMBC experiments.


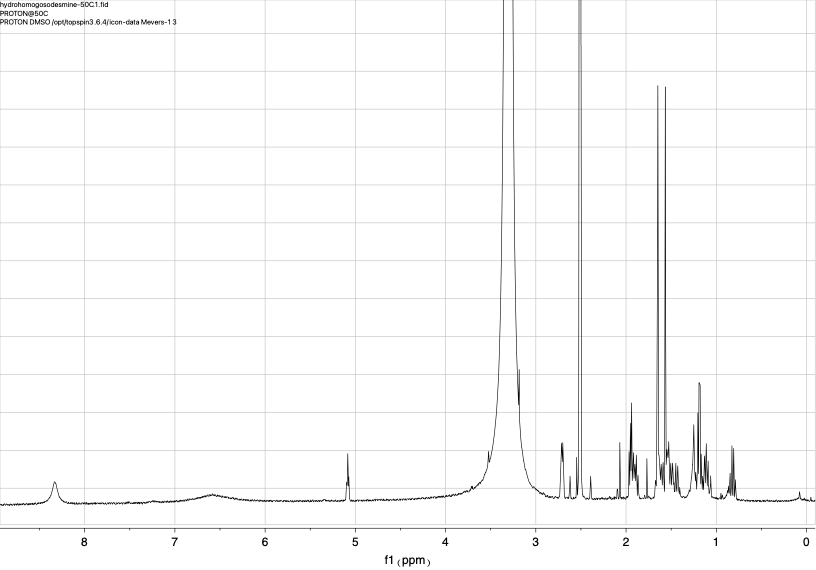


**Figure S45**. ^1^H NMR spectrum for homo-hydrogosodesmine (**6**) (600 MHz, *d_6_*-DMSO). 128 scans, 50^o^C


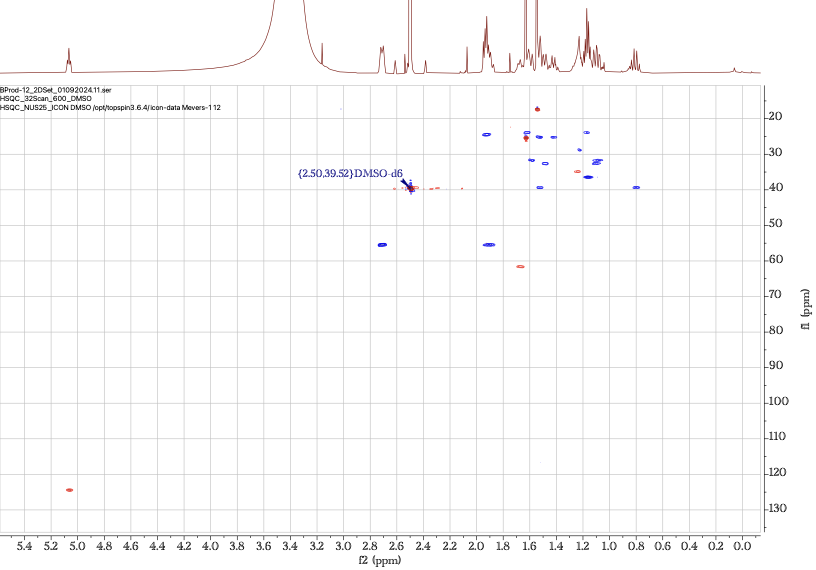


**Figure S46.** gHSQC for homo-hydrogosodesmine (**6**) (600 MHz, *d_6_*-DMSO). 32 scans, NUS25, 400 increments


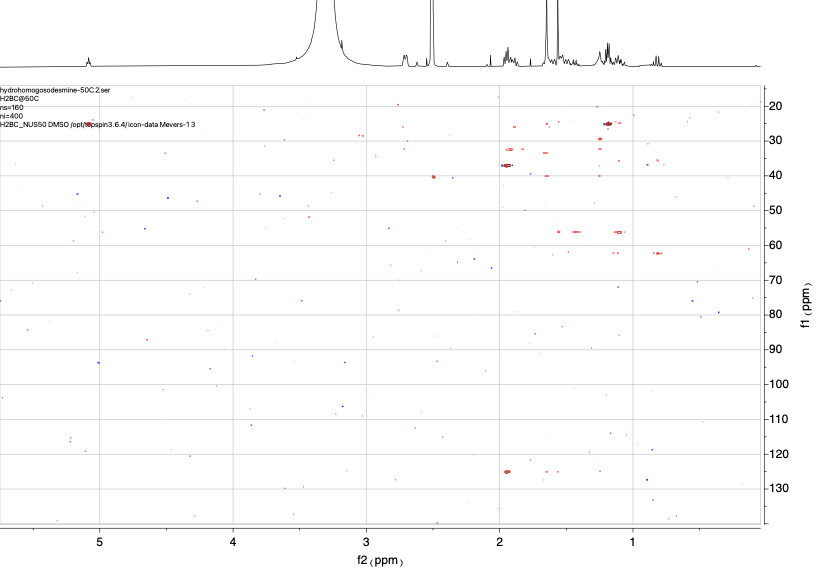


**Figure S47.** H2BC for homo-hydrogosodesmine (**6**) (600 MHz, *d_6_*-DMSO). 160 scans, NUS50, 400 increments, and 50^o^C


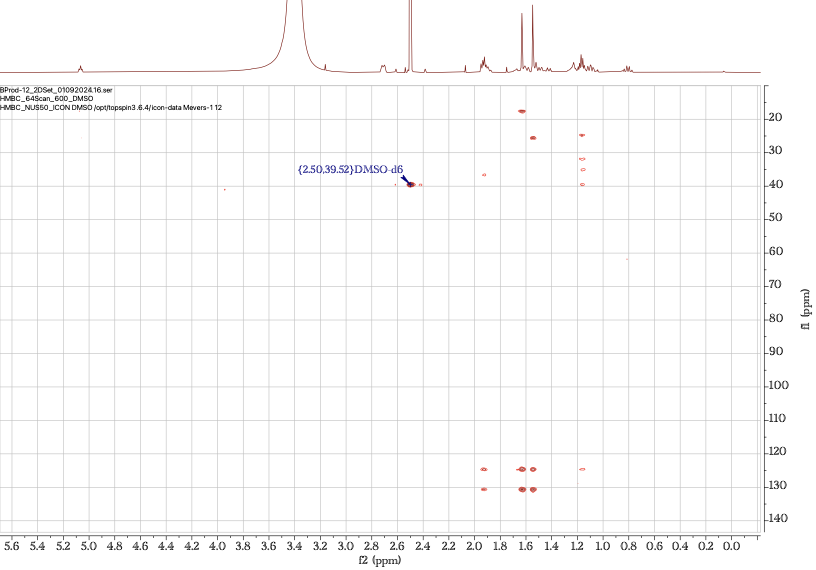


**Figure S48.** HMBC for homo-hydrogosodesmine (**6**) (600 MHz, *d_6_*-DMSO). 64 scans, NUS50, and 512 increments


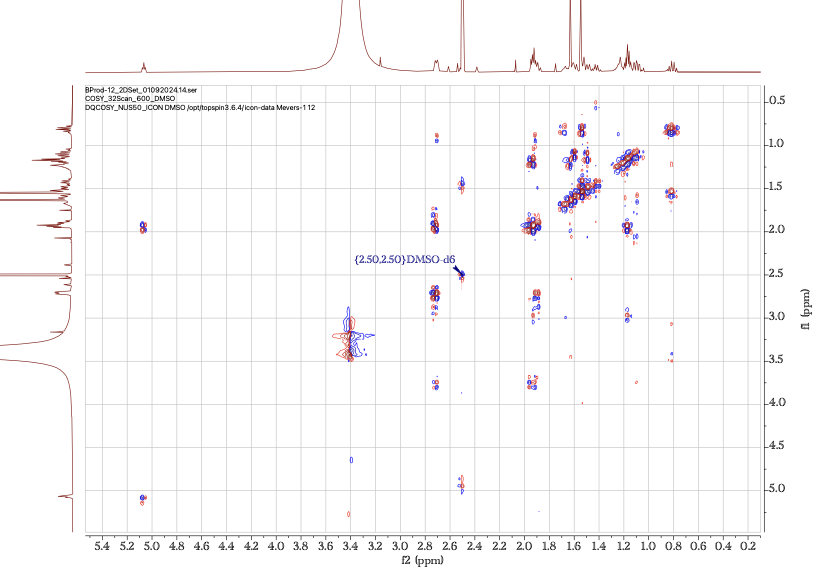


**Figure S49.** dqfCOSY for homo-hydrogosodesmine (**6**) (600 MHz, *d_6_*-DMSO). 32 scans, NUS50, and 400 increments.


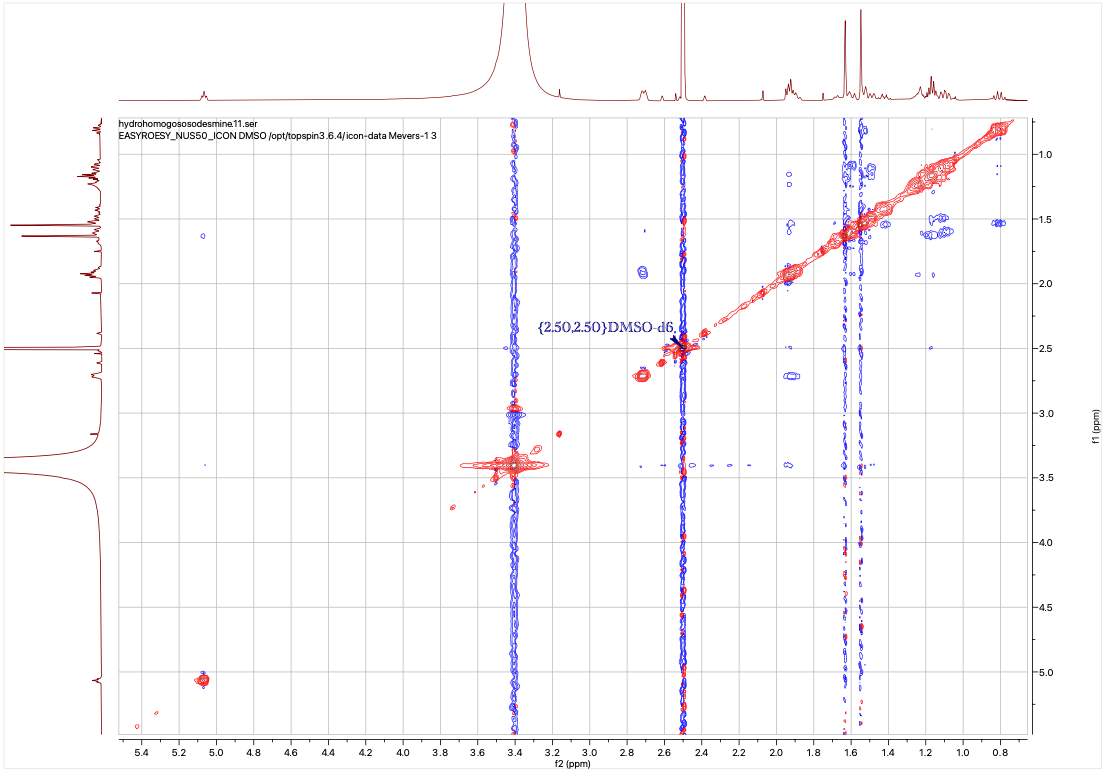


**Figure S50.** easyROESY for homo-hydrogosodesmine (**6**) (600 MHz, *d_6_*-DMSO). 128 scans, NUS50, 400 increments, and 400 μsec mixing time.
